# Depletion of protective microbiota promotes the incidence of fruit disease

**DOI:** 10.1093/ismejo/wrae071

**Published:** 2024-05-01

**Authors:** Xue Luo, Kai Sun, Hao-Ran Li, Xiang-Yu Zhang, Yi-Tong Pan, De-Lin Luo, Yi-Bo Wu, Hui-Jun Jiang, Xiao-Han Wu, Chen-Yu Ma, Chuan-Chao Dai, Wei Zhang

**Affiliations:** Jiangsu Key Laboratory for Microbes and Functional Genomics, Jiangsu Engineering and Technology Research Center for Industrialization of Microbial Resources, College of Life Sciences, Nanjing Normal University, Jiangsu Province, 210023, China; Jiangsu Key Laboratory for Microbes and Functional Genomics, Jiangsu Engineering and Technology Research Center for Industrialization of Microbial Resources, College of Life Sciences, Nanjing Normal University, Jiangsu Province, 210023, China; Jiangsu Key Laboratory for Microbes and Functional Genomics, Jiangsu Engineering and Technology Research Center for Industrialization of Microbial Resources, College of Life Sciences, Nanjing Normal University, Jiangsu Province, 210023, China; Jiangsu Key Laboratory for Microbes and Functional Genomics, Jiangsu Engineering and Technology Research Center for Industrialization of Microbial Resources, College of Life Sciences, Nanjing Normal University, Jiangsu Province, 210023, China; Jiangsu Key Laboratory for Microbes and Functional Genomics, Jiangsu Engineering and Technology Research Center for Industrialization of Microbial Resources, College of Life Sciences, Nanjing Normal University, Jiangsu Province, 210023, China; Jiangsu Key Laboratory for Microbes and Functional Genomics, Jiangsu Engineering and Technology Research Center for Industrialization of Microbial Resources, College of Life Sciences, Nanjing Normal University, Jiangsu Province, 210023, China; Jiangsu Key Laboratory for Microbes and Functional Genomics, Jiangsu Engineering and Technology Research Center for Industrialization of Microbial Resources, College of Life Sciences, Nanjing Normal University, Jiangsu Province, 210023, China; Jiangsu Key Laboratory for Microbes and Functional Genomics, Jiangsu Engineering and Technology Research Center for Industrialization of Microbial Resources, College of Life Sciences, Nanjing Normal University, Jiangsu Province, 210023, China; Jiangsu Key Laboratory for Microbes and Functional Genomics, Jiangsu Engineering and Technology Research Center for Industrialization of Microbial Resources, College of Life Sciences, Nanjing Normal University, Jiangsu Province, 210023, China; Jiangsu Key Laboratory for Microbes and Functional Genomics, Jiangsu Engineering and Technology Research Center for Industrialization of Microbial Resources, College of Life Sciences, Nanjing Normal University, Jiangsu Province, 210023, China; Jiangsu Key Laboratory for Microbes and Functional Genomics, Jiangsu Engineering and Technology Research Center for Industrialization of Microbial Resources, College of Life Sciences, Nanjing Normal University, Jiangsu Province, 210023, China; Jiangsu Key Laboratory for Microbes and Functional Genomics, Jiangsu Engineering and Technology Research Center for Industrialization of Microbial Resources, College of Life Sciences, Nanjing Normal University, Jiangsu Province, 210023, China

**Keywords:** Aspergillus, Bacillus, cropping conditions, fruit disease, jasmonic acid, peanut, synthetic microbial communities

## Abstract

Plant-associated microbiomes play important roles in plant health and productivity. However, despite fruits being directly linked to plant productivity, little is known about the microbiomes of fruits and their potential association with fruit health. Here, by integrating 16S rRNA gene, ITS high-throughput sequencing data, and microbiological culturable approaches, we reported that roots and fruits (pods) of peanut, a typical plant that bears fruits underground, recruit different bacterial and fungal communities independently of cropping conditions and that the incidence of pod disease under monocropping conditions is attributed to the depletion of *Bacillus* genus and enrichment of *Aspergillus* genus in geocarposphere. On this basis, we constructed a synthetic community (SynCom) consisting of three *Bacillus* strains from geocarposphere soil under rotation conditions with high culturable abundance. Comparative transcriptome, microbiome profiling, and plant phytohormone signaling analysis reveal that the SynCom exhibited more effective *Aspergillus* growth inhibition and pod disease control than individual strain, which was underpinned by a combination of molecular mechanisms related to fungal cell proliferation interference, mycotoxins biosynthesis impairment, and jasmonic acid–mediated plant immunity activation. Overall, our results reveal the filter effect of plant organs on the microbiome and that depletion of key protective microbial community promotes the fruit disease incidence.

## Introduction

Plant-associated microbiomes play important roles in plant fitness, development, and immunity [1, 2]. Dysbiosis of plant-associated microbiomes can result in the emergence and propagation of plant pathogens, leading to plant diseases [3–5]. For instance, long-term monocropping facilitates the outbreaks of soil-borne diseases by reducing the abundance of beneficial soil microbes and increasing pathogen abundance [6]. Disruption of protective Firmicutes and Actinobacteria abundance in the tomato rhizosphere promotes the incidence of bacterial wilt disease [3]. Similarly, dysbiosis of Firmicutes and Proteobacteria in the phyllosphere causes leaf necrosis and chlorosis [4]. These observations suggest that eubiosis of the microbiome is important for plant health. To date, knowledge on the microbiome and plant health has mostly been obtained from studies on plant vegetative organs such as roots and leaves. In comparison, little is known about the microbiome of plant reproductive organs [7]. Moreover, whether the knowledge gained from roots and leaves applies to plant reproductive organs, such as fruits, is largely unknown. Specifically, whether the occurrence of fruit disease can be attributed to dysbiosis of the fruit-associated microbiome remains to be verified and investigated.

Fruits, including seeds, represent the most crucial stages of a plant’s life history [8]. They are key components of plant fitness and are key to the sustainability of the agri-food system [9]. Moreover, unlike roots and leaves, which are present throughout a large part of the plant life cycle [7], fruits, as reproductive organs, develop on mature plants and are often present for a limited period. Consequently, research characterizing the microbiome of fruits has long lagged behind that on plant vegetative organs. Generally, fruits are rich in sugars, amino acids, polysaccharides, glycoproteins, and lipids, which provide an excellent niche for microbial colonization and offer an opportunity for pathogen infection [10–12]. For example, strawberry fruits and tomato tubers are susceptible to the phytopathogenic fungus *Botrytis cinerea*, the causative agent of gray mold disease [12]. *Aspergillus* species, especially *A. flavus* and *A. niger*, cause fruit rot in many plants, including peanut, maize, strawberry, and kiwifruit [13–15]. Despite cumulative laboratory and field experiments showing that fruits, whether underground or aboveground, are highly vulnerable to fungal pathogens, the mechanisms underlying the occurrence of fruit disease are largely unknown. In this regard, uncovering the microbial differences between plant vegetative and reproductive organs and the links between fruit health and microbial community is of considerable research interest.

In the past decade, an increasing number of studies have explored the role of beneficial microbes in plant disease control. Beneficial microbes protect plants from pathogen attacks by directly inhibiting the growth of pathogens and/or indirectly activating induced systemic resistance (ISR) [16, 17]. For example, *Streptomyces*, *Bacillus*, and *Pseudomonas* species inhibit soil-borne pathogens by producing the antibiotic lipopeptides [18]. ISR is primarily mediated by jasmonic acid (JA) and salicylic acid (SA) and can protect plants against a broad spectrum of pathogens [19]. However, research on the biocontrol of plant pathogens has focused on a limited number of individual microbial strains, which often generate inconsistent outcomes when applied in the field. Emerging evidence has indicated that plant defense can also be triggered by the entire microbiome or specific microbial consortia [20, 21]. Recently, studies have attempted to design synthetic microbial communities (SynComs) to mimic the microbiome under natural conditions [22, 23] and indicated that the SynComs are more effective in plant growth promotion and disease control under controlled and field conditions [24, 25]. Compared to single strains, SynComs show advantages in survival in soil conditions and interactions with plants. Thus, the introduction of SynComs is an important approach for elucidating the links between plant disease occurrence and the microbial community.

Here, we profiled the microbiomes from rhizosphere and geocarposphere of peanut (*Arachis hypogaea* L.) under monocropping (MP) and rotation (RP) cropping regimes and correlated the geocarposphere microbiome with pod disease incidence. Although previous studies have indicated the presence of distinct microbiota in the rhizosphere and phyllosphere [26–28], roots and leaves have different growth environment. Consequently, the rhizosphere and phyllosphere microbiomes can be influenced by different environmental factors. Specifically, in addition to the plant organ itself, the phyllosphere microbiota is affected by wind, insect visits, and water splashes [29, 30], whereas the rhizosphere microbiota is mainly affected by soil conditions, such as soil pH, structure, moisture, and nutrients [31]. Therefore, roots and leaves are not excellent models to specifically study the plant organ filter effect on the microbiome. A salient characteristic of peanut is aerial flowering and subterranean fruit [32]. After flowering and fertilization, peanut gynophores elongate to form a peg, and the peg-harboring embryo continues to grow and push the developing pod into the soil to develop underground pods [33]. As the roots and pods of peanut share the same soil environment, peanut is an appropriate model to investigate the filter effect of plant organs on the microbial community. The objectives of this study were to (i) explore whether roots and pods can recruit a specific microbiota, (ii) elucidate the interactions between pod disease incidence and the geocarposphere microbiome, and (iii) construct a SynCom derived from geocarposphere soil of healthy pods and to determine whether and how such a SynCom reduces pod disease.

## Materials and methods

### Plant materials and field experimental setup

**Figure 1:**
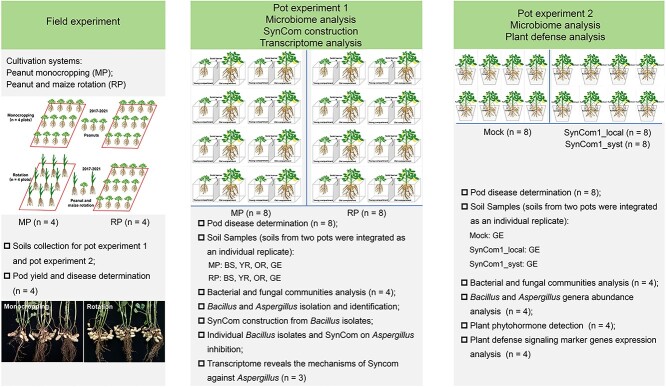
Schematic diagram of the key experimental arrangements in this study. (A) Field trial setup and sampling. The field trial was consisted of two different cultivation systems: (i) peanut under a monocropped (MP) regime and (ii) maize and peanut under a rotated (RP) regime. (B) Pot experiment 1 setup and sampling, microbiome analysis, SynCom construction, and fungal transcriptome analysis. (C) Pot experiment 2 setup and sampling, microbiome analysis, and plant defense analysis.

**Figure 2:**
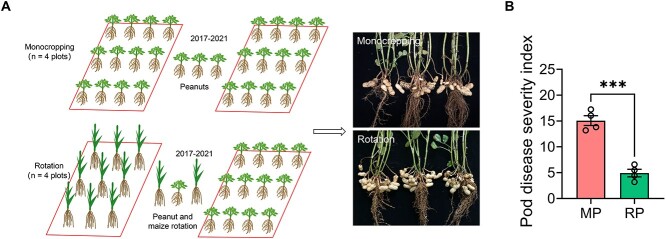
MP increases pod disease of peanut. (A) Field trial setup. Representative images of monocropped and rotated peanut plants. (B) Monocropping increases pod disease of peanut. Data are the mean ± SEM (*n* = 4 individual treatment). The asterisk indicates a significant difference between monocropped and rotated treatments according to Student’s *t* test (^*^^*^^*^P < 0.001). MP, monocropping; RP, rotation.

Peanut (Ganhua-5) and maize (Xianyu-335) seeds were used in the present study. The seeds were obtained from the Ecological Experimental Station of Red Soil, Chinese Academy of Science (Yintan, Jiangxi Province, China; 28°13’N, 116°55′E). The field trial was conducted at the Botanical Garden of Nanjing Normal University (Jiangsu Province, China; 32°3’N, 118°45’E) and consisted of two different cultivation systems: (i) peanut under a monocropped (MP) regime and (ii) maize and peanut under a rotated (RP) regime. The field was split into eight plots in the fall of 2017. Each treatment included four plots and each plot was 5 × 4 m (length × width) in size. The field experiment was set up in a randomized complete block design. For the MP regime, peanut plants were continuously grown for 4 years (2017–2020). For RP regime, the plots were cultivated with peanut in 2017 and 2019 and cultivated with maize in 2018 and 2020. The field trial was conducted in 2021 (Figs 1A and 2A). The peanut was sow in May and harvested in September. At the harvest, 10 peanut plants from each plot were collected. The number, mass, and disease status of the peanut pods were recorded. Pod disease was assessed by measuring the proportion of the total lesion area on the pod surface according to the following visual appearance scale: 0, no lesion; 1, lesion area < 1/4; 2, 1/4 ≤ lesion area < 1/2; 3, 1/2 ≤ lesion area < 3/4; and 4, lesion area ≥ 3/4. The disease index was calculated as Σ (disease scale/the highest scale × proportion of corresponding pod within each class) [34].

### Pot experiment 1: setup and sampling

**Figure 3:**
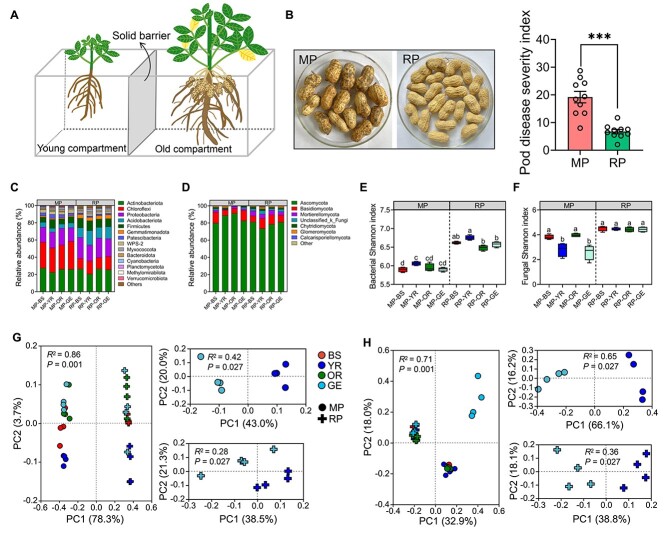
MP changes microbial diversity and composition. (A) Pot experiment 1 setup. (B) Monocropping increases pod disease of peanut. Representative images of monocropped and rotated peanut pods in the old compartment. Data are the mean ± SEM (*n* = 8 individual replicates). The asterisk indicates a significant difference between monocropped and rotated treatments according to Student’s *t* test (^*^^*^^*^P < 0.001). (C, D) Relative abundance of bacterial (C) and fungal (D) phyla in MP-BS, MP-YR, MP-OR, MP-GE, RP-BS, RP-YR, RP-OR, and RP-GE samples. **(**E, F) Bacterial (E) and fungal (F) Shannon index in MP-BS, MP-YR, MP-OR, MP-GE, RP-BS, RP-YR, RP-OR, and RP-GE samples. Boxplots indicate median (middle line), 25th, 75th percentiles (box), and maximum and minimum values (whiskers) (*n* = 4 individual replicates). Different letters indicate significant differences among treatments (^*^P < 0.05, one-way analysis of variance followed by Tukey’s honest significant difference test). (G) PCoA (based on the relative abundance of bacterial OTUs) of Bray–Curtis distances of MP-BS, MP-YR, MP-OR, MP-GE, RP-BS, RP-YR, RP-OR, and RP-GE samples. (H) PCoA (based on the relative abundance of fungal OTUs) of Bray–Curtis distances of MP-BS, MP-YR, MP-OR, MP-GE, RP-BS, RP-YR, RP-OR, and RP-GE samples. PERMANOVA was performed using the adonis function from the R package. MP, monocropping; RP, rotation; MP-BS, monocropped-bulk soil; MP-YR, monocropped-young rhizosphere soil; MP-OR, monocropped-old rhizosphere soil; MP-GE, monocropped-geocarposphere soil; RP-BS, rotated-bulk soil; RP-YR, rotated-young rhizosphere soil; RP-OR, rotated-old rhizosphere soil; RP-GE, rotated-geocarposphere soil.

Pot experiment 1 included two treatments: MP and RP. The soils (0–20 cm) were collected from the MP and RP plots in April 2021 before the field trial. For each plot, two square plastic pots (length × width × height = 40 × 30 × 30 cm) were established. A total of 16 pots were included, and each treatment contained 8 pots. Each pot was separated into young and old compartments by a solid barrier (Figs 1B and 3A). A peanut seed was first sown into the old compartment. When the peanut pegs were formed and penetrated into the soil (60 days after sowing), a new peanut seed was sown into the young compartment. Thus, the pods in the old compartment and roots in young compartment shared similar growth and development time underground. The pots were randomly placed in a greenhouse (day: 25–30°C; night: 20–25°C, 60 ± 5% relative humidity). At 30 days after new seed sowing, the bulk soil (BS) and rhizosphere soil (YR) was collected from the young compartment; rhizosphere soil (OR), geocarposphere soil (GE), and pods were collected from the old compartment. The soils from two pots in each treatment were integrated as a sample; thus, four individual replicates were set up for physicochemical properties and microbiome analyses. The pods were used for disease analysis. The soil physicochemical properties are provided in Table S1.

### Soil DNA extraction, 16S rRNA amplicon sequencing, ITS amplicon sequencing, and bioinformatics analysis

Soil samples (0.5 g) from the pot experiment were extracted using the FastDNA SPIN Kit (MP Biomedical, Irvine, CA, USA) according to the manufacturer’s instructions. The concentration and integrity were confirmed by NanoDrop spectrophotometry (Thermo) and electrophoresis, respectively. Amplicon libraries were prepared using tagged universal primers for bacteria (338F/806R) [35] and fungi (ITS1/ITS2) [36]. Each sample was amplified in a 20-μl reaction system. The reaction system included 0.5-μM forward and reverse primers, 1 × Premix Taq DNA polymerase (Takara, Kusatsu, Japan), and 20 ng of DNA templates. The amplification was carried out under the following conditions: initial denaturation at 95°C for 3 min, 20 cycles of denaturation at 95°C for 30 s, annealing at 55°C for 30 s, and extension at 72°C for 5 min and a final extension at 72°C for 1 min. Amplicon sequencing libraries were constructed using the MiSeq Reagent Kit v3. Paired-end 300-bp reads were sequenced on a MiSeq platform (Illumina) according to standard protocols.

The raw data were screened and trimmed by the QIIME pipeline, and paired-end sequences were merged using Flash [37, 38]. The sequences were then clustered into operational taxonomic units (OTUs) at a pairwise identity threshold of 97% using UPARSE [39]. Each OTU was taxonomically annotated by using the RDP Classifer (2.13) and bacterial silva (138) and fungal ITS UNITE (8) databases. Principal covariate analysis (PCoA) was performed using the Bray–Curtis dissimilarity matrix with the vegan package in R to explore patterns of bacterial and fungal community composition. Differences in bacterial and fungal community composition across treatments were determined with permutational multivariate analysis of variance (PERMANOVA) using the adonis function from R package [40]. Linear discriminant analysis (LDA) of effect size (LEfSe) was applied to the OTU table to identify the differentially abundant taxa among treatments [41]. The nonparametric factorial Kruskal–Wallis (KW) sum-rank test (*P* < 0.05) and the absolute LDA score (>3.7) were used to analyze the statistical significance and strength, respectively. The raw sequencing data were deposited using the SRA service of the GenBank database under the accession number PRJNA989386, PRJNA992791, PRJNA989419, and PRJNA989426.

### Isolation and identification of *Bacillus* and *Aspergillus* from the geocarposphere

Geocarposphere soils from the RP and MP treatments in the pot experiment were collected for the isolation of *Bacillus* and *Aspergillus*, respectively. The soil was filtered through a 2-mm mesh to remove large soil particles and suspended in sterile phosphate-buffered saline (PBS, pH 7.2). The suspension was shaken at 180 rpm for 30 min. Briefly, the suspension was incubated at 80°C for 30 min and then spread on V8 *Bacillus* semiselective medium after serial dilution and incubated at 30°C for 5 days [42]. One representative of each single colony was selected according to the bacterial morphology. Bacterial colonies were purified and stored at −80°C in 20% glycerol until further use. The identification of isolates was based on 16S rRNA gene sequencing with the primer pair 27F/1492R. The obtained 16S rRNA gene sequences were blasted against the National Central for Biotechnology Information (NCBI) database to identify homologous sequences, and the closest match was identified. The 16S rRNA sequences have been deposited in the GenBank database under accession numbers OQ875794–OQ875804. The *Bacillus* strains were deposited in 50% glycerin solutions and stored in −80°C ultra-low temperature refrigerator (Thermo Scientific, Waltham, MA, USA).

The isolation of *Aspergillus* was performed according to a modified protocol [43]. Briefly, the suspension was plated on potato dextrose agar (PDA) medium supplemented with antibacterial agents, streptomycin (20 μg/ml) and penicillin (20 μg/ml) after serial dilution, and incubated at 28°C for 7 days. Based on fungal morphology, one representative of each single colony was selected. The identification of isolates was based on 18S rRNA gene sequencing with the primer pair ITS1/ITS4. The obtained ITS sequences were blasted against the NCBI database to identify homologous sequences, and the closest match was identified. The ITS sequence reads have been deposited in the GenBank database under accession numbers from OQ874532 to OQ874540. The *Aspergillus* strains were deposited in 30% glycerin solutions and stored in −80°C ultra-low temperature refrigerator (Thermo Scientific, Waltham, MA, USA).

### Evaluation of *Aspergillus* suppression by *Bacillus* isolates

To test the antagonistic activity of *Bacillus* isolates against *Aspergillus flavus* GE1 and *A. niger* GE2, a dual culture assay on TSA medium was performed. To this end, an *Aspergillus*-colonized PDA plug (5-mm diameter) was placed on one side of a petri dish (Ø 9 cm) with TSA. *Bacillus* isolates were then streaked on the other side, at an initial 30 mm away from the *Aspergillus* plug. The petri dishes were then sealed with Parafilm and incubated at 28°C for 5 days. Plates with only *A. flavus* or *A. niger* inoculation alone were also included as controls. The antagonistic activity (%) was calculated using the following equation: [1-(Aa-Ap)/Aa] × 100, where Aa is the area of hyphal growth in the absence of *Bacillus* isolates, and Ap is the area of fungal growth in the presence of *Bacillus* isolates [44]. The area of fungal growth was determined using ImageJ software (National Institutes of Health, Bethesda, MD, USA). Ten individual replicates were performed.

The antagonistic activity of *Bacillus* isolates against *A. flavus* GE1 and *A. niger* GE2 on detached pods was detected. Pods were surface-sterilized by immersion in 70% (v/v) ethanol for 1 min and 1.5% (v/v) sodium hypochlorite solution for 15 min, followed by three rinses with sterile water. To check if the pods were well surface-sterilized, the pods and 100 μl of the remaining washing water were placed on TSA and PDA plates. Pods having no colony growth will be used for downstream treatment. Each pod was coinoculated with 1 ml of spore suspension (1 × 10^7^ conidia ml^−1^) and 1 ml of *Bacillus* isolates, SynCom1 or SynCom2. The pods were then placed on plates, and each plate contained five pods. To prepare the *Bacillus* isolates, the selected *Bacillus* strains were cultured in TSB medium for 24 h and centrifuged for 10 min at 4000 g. After three washes with sterile water, bacterial concentration was adjusted to OD_600_ = 0.6 in sterile water. The SynCom1 consisted of three *Bacillus* strains, *B. cereus* GE1, *B. amyloliquefaciens* GE2, and *B. altitudinis* GE3, which exhibited high culturable abundance (43.90% for BcGE1, 21.95% for BaGE2, and 9.76% for BaGE3) in the 41 isolated *Bacillus* strains. The SynCom2 comprised *B. halotolerans* GE7, *Paenibacillus* sp. GE8, and *B. siamensis* GE10, which were randomly selected from the isolated *Bacillus* strains with culturable abundance <5%. SynCom2 was set up as a control of SynCom1. To prepare the SynCom1 and SynCom2, equal volumes of corresponding *Bacillus* suspension cultures were mixed [45], and then the OD_600_ of SynCom was adjusted to 0.6. Each treatment was performed in five to six individual replicates. After incubation at 28°C for 5 days, the pods were collected for *Aspergillus* biomass quantification. Specific primer pairs of *A. flavus* GE1 (Fla-F/Fla-R) [46] and *A. niger* GE2 (An-F/An-R) [47] were used to quantify the *Aspergillus* amount on the surface of pods with quantitative PCR with a 7500 Real-Time PCR System (Applied Biosystems, Pleasanton, CA, USA). Gene copy numbers were expressed as log_10_ values. The primers are listed in Table S2.

### RNA-sequencing and bioinformatics analysis

An *Aspergillus*-colonized PDA plug (5-mm diameter) was placed on one side of a petri dish (Ø 9 cm) with TSA medium. SynCom1 was then streaked on the other side, at an initial 30 mm away from the *Aspergillus* plug. *A. flavus* or *A. niger* inoculation alone without SynCom1 were included as controls. The petri dishes were previously covered with a transparent cellophane sheet to facilitate the collection of mycelia. After incubated at 28°C for 5 days, mycelia of *A. flavus* and *A. niger* were collected from plates for RNA-Seq analysis. Three individual replicates were performed. Total RNA was extracted with TRIzol reagent (Invitrogen). The concentration and integrity were confirmed with NanoDrop spectrophotometry (Thermo, Waltham, MA, USA) and electrophoresis, respectively. RNA was used for RNA-Seq library construction according to the manufacturer’s instructions (Illumina, San Diego, CA, USA). The short insert library was sequenced on the HiSeq X Ten platform following the manufacturer’s protocols. The resulting reads were aligned to the reference genomes of *A. flavus* NRRL3357 (https://www.ncbi.nlm.nih.gov/genome/360?genome_assembly_id=968150) and *A. niger* NRRL3_1 (https://mycocosm.jgi.doe.gov/Aspni_NRRL3_1/Aspni_NRRL3_1.home.html). Differentially expressed genes (DEGs) were identified with a *P* < 0.05 and fold change >2 or fold change <0.05 as thresholds using the “DESeq2” package. Gene Ontology (GO) enrichment and Kyoto Encyclopedia of Genes and Genomes (KEGG) pathway enrichment of DEGs were performed using R. The raw sequencing data were deposited using the SRA service of the GenBank database under the accession number PRJNA989718 and PRJNA989723.

### Pot experiment 2: setup and sampling

Pot experiment 2 was set up to determine whether SynCom1 has the ability to locally and systemically reduce pod diseases under MP conditions. The soils (0–20 cm) for pot experiment 2 were collected from the MP plots in April 2021 before the field trial. Peanut seedlings were grown in pots (28-cm diameter, 22-cm height). When the peanut pegs were formed and began to penetrate the soil (60 days after sowing), an Erlenmeyer flask (50 ml) with MP soil was inserted into the pots to allow peg growth. Each Erlenmeyer flask contained one peg, and each pot included two Erlenmeyer flasks. Pot experiment 2 contained the following treatments: (i) control, Erlenmeyer flask with 1 ml of sterile water inoculation; (ii) Syncom1_local, Erlenmeyer flask with 1 ml of SynCom1 suspension inoculation; and (iii) Syncom1_systemic, Erlenmeyer flask with 1 ml of sterile water inoculation (Figs 1C and 8A). To conduct the SynCom1 treatment, equal volumes of BcGE1, BaGE2, and BaGE3 suspension cultures were mixed [45], and then the OD_600_ of SynCom1 was adjusted to 0.6. Each treatment contained eight pots. Syncom1_local and Syncom1_systemic treatments were established on the different pods of same peanut plant. Thus, a total of 16 pots were established. At 30 days after inoculation, GE samples and pods were collected. The soils were used for microbiome analysis, and pods were used for disease determination, plant phytohormone detection, and defense signaling marker gene analyses. The soils from two pots in each treatment were integrated as a sample; thus, four individual replicates were set up for microbiome analysis. After pod disease determination, the pods from two pots in each treatment were integrated as a sample for plant phytohormone detection and defense signaling marker gene analyses. Thus, eight individual replicates were set up for pod disease determination, and four and four individual replications were set up for plant phytohormone detection and defense signaling marker gene analyses, respectively.

### Plant phytohormone detection

JA, SA, and abscisic acid (ABA) were extracted from the pods and quantified by high-performance liquid chromatography (HPLC) after extraction, purification, and filtration (0.22 μm) according to a modified protocol [48, 49]. Briefly, 150 mg of fresh pod shells was ground into powder in liquid nitrogen and extracted with 1.0 ml of methanol:formic acid:water (79:20:1, v/v/v) overnight at 4°C. The suspension was centrifuged at 12 000 rpm for 30 min at 4°C, and the solid residue was re-extracted and recentrifuged, and the supernatants were pooled. The supernatants were then passed through an anion-exchange column and dried with nitrogen gas. The residue was dissolved in 150-μl methanol. JA, SA, and ABA were quantified using an LC–MS/MS system with a C18 column (Agilent Technologies, USA) with 0.05% formic acid (A) and methanol (B) as the mobile phase at a flow rate of 0.3 ml min^−1^. The column temperature was maintained at 40°C, and the injection volume was 10 μl. The JA, SA, and ABA calibration standards were processed at concentrations of 0.1, 1.0, 5.0, 10.0, 20.0, 40.0, 80.0, and 100.0 ng ml^−1^. The levels of JA, SA, and ABA were calculated based on the standard curves in units of nanogram per milligram fresh weight. Four individual replicates were performed.

### Expression analysis of defense signaling marker genes

Total RNA was extracted from peanut pods with TRIzol reagent (Vazyme Biotech Co., Ltd.) according to the manufacturer’s instructions. The RNA was inoculated with DNase I to remove genomic DNA, and first-strand cDNA was generated with a Reverse Transcription System Kit (Vazyme Biotech Co., Ltd.). Quantitative real-time PCR (RT-PCR) was carried out on a 7500 Real-Time PCR System (Applied Biosystems, Pleasanton, CA, USA) using AceQ qPCR SYBR Green Master Mix (Vazyme Biotech Co., Ltd.). Peanut *Actin* was used as a reference gene [50]. The relative expression of target genes was determined by the comparative Ct method. The experiment was carried out in four independent replicates. The primers are listed in Table S2.

### Statistical analysis

All experiments were performed at least three individual replicates. The data were expressed as the mean with standard error (SE). The data were analyzed with SPSS 18.0 (SPSS lnc., Chicago, IL, USA). Significant differences were determined with one-way ANOVA followed by Tukey’s multiple honest significant difference (HSD) test (*P* < 0.05) or two sided Student’s *t-*test (^*^P < 0.05; ^*^^*^P < 0.01; ^*^^*^^*^P < 0.001). Correlations among *Bacillus* relative abundance, *Aspergillus* relative abundance, and pod disease severity index were performed by Pearson correlation analysis.

## Results

### MP reduces pod set and increases pod disease

To explore the effects of MP on pod productivity and disease, a field trial consisting of MP and RP regimes was set up (Fig. 2A). Compared to RP, MP significantly reduced pod number and pod weight per plant by 32.7% and 37.1%, respectively (Fig. S1A and B). In contrast, no significant difference in weight per pod was found between the MP and RP regimes (Fig. S1C). Thus, the reduced pod weight in MP was due to the decline in pod formation. Moreover, MP increased the disease severity of pods by 67.3% (Fig. 2B).

### MP changes microbial diversity and composition

To ensure that the pods and roots shared the similar growth and development time underground, young and old compartments with MP- and RP-conditioned soil were set up in the pot experiment. A new peanut seed was sown in the young compartment when the peanut pegs in the old compartment began to penetrate the soil (Fig. 3A). Consequently, at the time of the experiment, the pods in the old compartment and roots in the young compartment shared similar times underground. Consistent with the field trial, pods in the old compartment cultivated with MP-conditioned soil showed higher levels of disease than those cultivated with RP-conditioned soil (Fig. 3B). At 30 days after new seed sowing, we collected bulk soil (BS) and rhizosphere soil (YR) from the young compartment and rhizosphere soil (OR) and geocarposphere soil (GE) from the old compartment for 16S rRNA and ITS sequencing.

The results of bacterial community analysis revealed that Actinobacteriota, Chloroflexi, Proteobacteria, Acidobacteriota, and Firmicutes were the top five major bacterial taxa at the phylum level (Fig. 3C). MP significantly increased the abundance of Chloroflexi but significantly reduced the abundance of Acidobacteriota. When comparing BS and GE samples, we found no significant difference in the abundance of the top five bacterial communities at the phylum level. When comparing YR, OR, and GE samples, we found that the abundance of Actinobacteriota in GE was similar to that in OR, but was higher than that in YR independent of cropping system (Table S3). In terms of fungi, Ascomycota, Basidiomycota, Mortierellomycota, Unclassified_k_Fungi, and Chytridiomycota were the top five major fungal taxa at the phylum level (Fig. 3D). MP increased the abundance of Ascomycota and reduced the abundance of Mortierellomycota. Compared to BS, GE samples showed an increased abundance of Ascomycota under MP conditions and an increased abundance of Basidiomycota under RP conditions. Under RP conditions, YR samples showed an increased abundance of Unclassified_k_Fungi (Table S4). Based on the Shannon and Chao1 indices, MP reduced bacterial alpha diversity compared to RP (Fig. 3E; Table S5). Meanwhile, MP reduced fungal alpha diversity in YR and GE compared to other treatments (Fig. 3F). Moreover, no significant difference in the Shannon and Chao1 indices was found between YR and GE under both MP and RP conditions (Fig. 3E and F; Fig. S2; Table S6).

Principal coordinates analysis (PCoA) revealed that significant differences in bacterial and fungal community composition were found among the samples from BS, YR, OR, and GE under MP and RP conditions (PERMANOVA, bacteria: *R*^2^ = 0.86, *P* = .001; fungi: *R*^2^ = 0.71, *P* = .001) (Fig. 3G and H). When classifying the samples into MP and RP based on cropping conditions, we found clear differences in bacterial and fungal communities between MP and RP conditions (Fig. S3), suggesting that the cropping regimes had a significant effect on microbiome composition. There were differences in the bacterial and fungal communities between the YR and OR samples (Fig. S4). In addition to the bacterial community under RP conditions, the bacterial and fungal communities of the BS and GE samples were separated (Fig. S5). Inspection of samples from YR and GE indicated that the bacterial and fungal communities in these two groups were clearly separated, irrespective of cropping conditions (Fig. 3G and H). Moreover, the bacterial and fungal communities of the OR and GE samples were separated, except for the bacterial communities between OR and GE under RP conditions (Fig. S6).

### Root and pod recruit distinct bacterial and fungal communities

**Figure 4:**
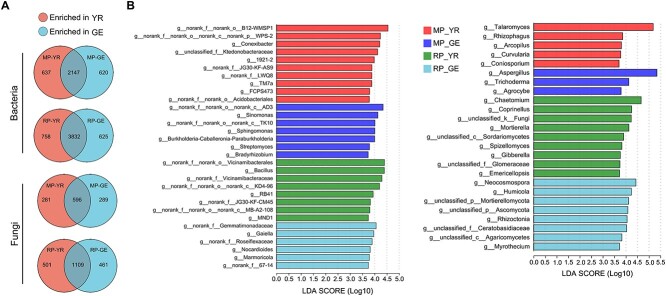
Root and pod recruit distinct bacterial and fungal communities. (A) Venn diagrams showing the overlap of bacterial and fungal OTUs in YR and GE samples under monocropping and rotation conditions. (B) Linear discriminant analysis (LDA) scores to identify the bacterial and fungal genera in YR and GE samples under monocropping and rotation conditions by LDA of the effect size (LEfSe). Only the taxa with an absolute LDA score > 3.7 are shown. YR, young rhizosphere soil; GE, geocarposphere soil.

To further probe into the differences in microbial features between roots and pods, we first examined root and pod-associated bacteria and fungi at the OTU level. The bacterial OTUs enriched accounted for 22.4% (620 out of 2767 OTUs) in GE and 22.9% (637 out of 2784 OTUs) in YR under MP conditions and for 14.0% (625 out of 4457 OTUs) in GE and 16.5% (758 out of 4590 OTUs) in YR under RP conditions. The fungal OTUs enriched accounted for 32.7% (289 out of 885 OTUs) in GE and 32.0% (281 out of 877 OTUs) in YR under MP conditions and for 29.4% (461 out of 1570 OTUs) in GE and 31.1% (501 out of 1610 OTUs) in YR under RP conditions (Fig. 4A; Table S7). Overall, the rhizosphere and geocarposphere comprised more generalists than specialists, as most bacterial and fungal OTUs were detected in both rhizosphere and geocarposphere soils.

To identify the bacterial and fungal genera with the significant influence on the difference in the microbiota between the YR and GE under MP and RP conditions, we performed LEfSe analysis. Under both MP and RP conditions, the LEfSe analysis (LDA score > 3.7, *P* < 0.05, KW sum-rank test) showed that different bacterial and fungal genera contributed to the changes in YR and GE microbiota, and more bacterial and fungal genera were significantly enriched in YR than in GE (Fig. 4B). The genera *Sphingomonas* (LDA score, 4.01), *Streptomyces* (3.79), and *Bradyrhizobium* (3.74), which commonly exhibit antifungal activity and plant growth promotion, and the genus *Aspergillus* (5.35), which causes fruit disease in many crops, were enriched in GE under MP conditions (Fig. 4B). Together, these results suggest that roots and pods recruit distinct microbial communities.

### MP decreases the abundance of *Bacillus* and increases the abundance of *Aspergillus* in the geocarposphere

**Figure 5:**
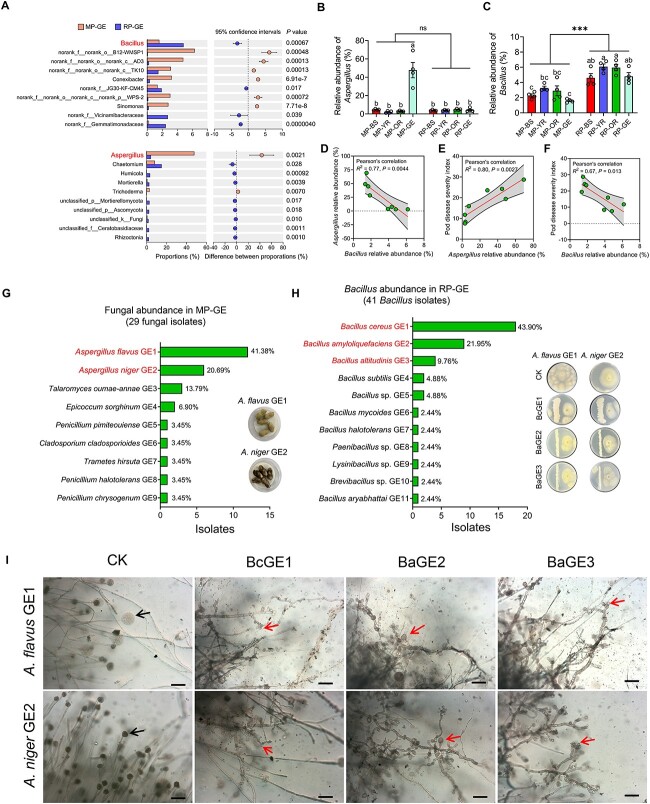
MP decreases the abundance of *Bacillus* and increases the abundance of *Aspergillus* in the geocarposphere*.* (A) Relative abundance of bacterial and fungal genera in MP-GE and RP-GE samples. Only the top 10 bacterial and fungal genera are shown. (B, C) Relative abundance of *Bacillus* (B) and *Aspergillus* (C) in MP-BS, MP-YR, MP-OR, MP-GE, RP-BS, RP-YR, RP-OR, and RP-GE samples. Data are the mean ± SEM (*n* = 4 individual replicates). Different letters indicate significant differences among treatments (^*^P < 0.05, one-way analysis of variance followed by Tukey’s honest significant difference test) and asterisk indicates a significant difference between monocropped and rotated treatments according to Student’s *t* test (^*^^*^^*^P < 0.001). ns indicates a nonsignificant difference. (D–F) pairwise correlation analyses among *Bacillus* abundance, *Aspergillus* abundance, and pod disease. (G) Isolation of fungi from MP-GE samples. Representative images showing pod diseases caused by *A. flavus* GE1 and *A. niger* GE2 inoculation. (H) Isolation of *Bacillus* from RP-GE samples. Representative images showing the antagonism of BcGE1, BaGE2, and BaGE3 against *A. flavus* GE1 and *A. niger* GE2 on TSA plates. (I) Images showing the morphology of hyphae proximal and distal to the BcGE1, BaGE2, and BaGE3. The arrows in CK treatment indicate conidiophores; the arrows in BcGE1, BaGE2, and BaGE3 treatments indicate the club-shaped morphology of hyphae; bars, 10 μm.

We hypothesized that the incidence of pod disease is attributed to the dysbiosis of the microbial community in GE. We thus focused on bacterial and fungal communities in GE samples under MP and RP conditions. The genus *Bacillus* was significantly enriched in RP-GE, whereas the genus *Aspergillus* was significantly enriched in MP-GE (Fig. 5A). Compared to RP, MP significantly increased the abundance of the genus *Aspergillus* (from 4.3% to 47.8%) (Fig. 5B) and reduced the abundance of the genus *Bacillus* (from 4.8% to 1.6%) in GE (Fig. 5C). Moreover, no significant difference in *Bacillus* abundance was found between GE and YR within the same cropping conditions (Fig. 5C), indicating that cropping regimes were the determinant for the decline in *Bacillus* abundance of GE samples. In contrast, *Aspergillus* was specifically enriched in MP-GE samples (Fig. 5B). The abundance of *Bacillus* was negatively correlated with *Aspergillus* (*R*^2^ = 0.77, *P* = .0044) (Fig. 5D). Additionally, *Aspergillus* abundance was positively correlated with pod disease (*R*^2^ = 0.80, *P* = .0027) (Fig. 5E), whereas *Bacillus* abundance was negatively correlated with pod disease (*R*^2^ = 0.67, *P* = .013) (Fig. 5F). These results indicate a role of *Bacillus* in controlling *Aspergillus* accumulation and reducing pod disease. In addition, we cannot exclude the potential contributions of other bacterial genera to the increased pod diseases under MP. For instance, six bacterial genera were enriched under MP, and their abundances were significantly positively correlated with *Aspergillus* abundance (Fig. 5A, Fig. S7). Previous studies had reported that some bacteria promote plant diseases by acting as pathogen helpers [51]. Whether these six enriched genera contributed to pod diseases as *Aspergillus* helpers is unknown. It is therefore possible that apart from the decreased abundance of *Bacillus*, the enrichment of some bacterial genera might contribute to the increased pod diseases under MP conditions.

### SynCom1 is more effective in inhibiting *Aspergillus* growth than individual strains

To further probe into the functions of *Bacillus* in reducing *Aspergillus* accumulation and pod disease, we isolated *Aspergillus* strain from MP-GE and *Bacillus* strain from RP-GE samples. Twenty-nine fungal isolates were obtained from MP-GE samples. The isolates belonged to *Aspergillus*, *Talaromyces*, *Epicoccum*, *Penicillium*, *Cladosporium*, and *Trameters* (Fig. 5G). We next inoculated pods with the fungal isolates to determine whether they could cause pod disease. Based on *in vitro* and *in vivo* experiments, inoculation with *A. flavus* GE1 and *A. niger* GE2 clearly caused pod disease (Fig. 5G; Figs S8 and S9). Considering that *A. flavus* GE1 and *A. niger* GE2 showed high culturable abundance (>20%; 41.38% and 20.69%, respectively) (Fig. 5G), *A. flavus* GE1 and *A. niger* GE2 were selected as key fungal pathogens in pods. Forty-one *Bacillus* isolates were obtained from RP-GE samples. Among the *Bacillus* isolates, *Bacillus cereus* GE1 (BcGE1), *B. amyloliquefaciens* GE2 (BaGE2), and *B. altitudinis* GE3 (BaGE3) were selected as keystone taxa isolates, as their culturable abundance was >5% (43.90%, 21.95%, and 9.76%, respectively) (Fig. 5H). The 16S rRNA gene of BcGE1, BaGE2, and BaGE3 showed 100% homology to OTU4532, OTU3766, and OTU4000, respectively (Fig. S10A). Compared to the MP-GE samples, the relative abundance of OTU3766 was significantly higher in RP-GE samples (*P* = .015). Although no significant difference in the relative abundance of OTU4532 and OTU4000 was found between RP-GE and MP-GE samples, the mean values of relative abundance of OTU4532 and OTU4000 were larger in RP-GE samples than those in MP-GE samples (Fig. S10B). When the isolated *Aspergillus* and *Bacillus* were coinoculated on tryptic soy broth agar (TSA) medium, we found that these three *Bacillus* isolates showed direct antagonism against *A. flavus* GE1 and *A. niger* GE2 (Fig. 5H; Fig. S11). Microscopic inspection found that hyphae of *A. flavus* GE1 and *A. niger* GE2 proximate to *Bacillus* stream were characterized by twisted, dichotomous branching and club-shaped morphology. Moreover, the *Bacillus* isolates inhibited the conidiophores formation of proximate hyphae (Fig. 5I).

**Figure 6:**
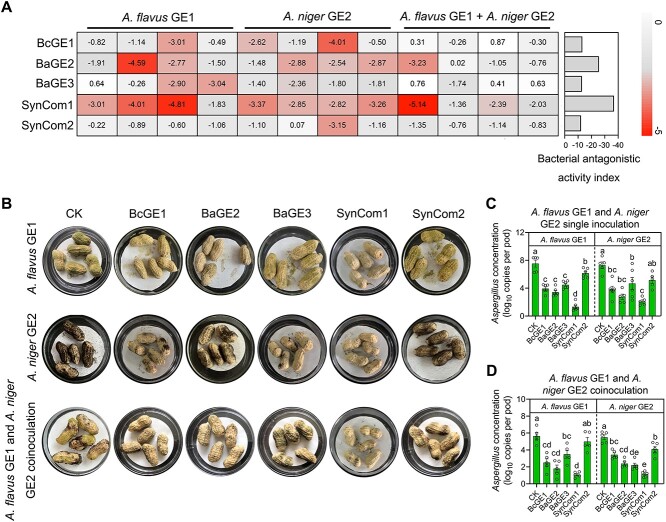
SynCom1 is better at inhibiting *Aspergillus* growth than single *Bacillus* strain. (A) Effects of BcGE1, BaGE2, BaGE3, SynCom1 (BcGE1 + BaGE2 + BaGE3), and SynCom2 (*B. mycoides* GE6 + *Paenibacillus* sp. GE8 + *B. siamensis* GE10) on the growth of *A. flavus* GE1 and *A. niger* GE2. The heatmap depicts the log2 fungal relative growth index (presence vs. absence of single strains or SynCom) measured by the WGA Alexa Fluor 488 conjugate. The horizontal bar plot indicates the cumulative antagonistic activity of the single strain and SynCom against *flavus* GE1 and *A. niger* GE2. (B) Representative images showing the *A. flavus* GE1 and *A. niger* GE2 on pods with sterile water, BcGE1, BaGE2, BaGE3, SynCom1 and SynCom2 coinoculation. (C, D) Quantification of *A. flavus* GE1 (C) and *A. niger* GE2 (D) biomass on pods with sterile water, BcGE1, BaGE2, BaGE3, SynCom1, and SynCom2 coinoculation. Data are the mean ± SEM (*n* = 5–6 individual replicates). Different letters indicate significant differences among treatments (^*^P < 0.05, one-way analysis of variance followed by Tukey’s honest significant difference test). SynCom, synthetic microbial community.

Construction of the SynComs is an essential step for elucidating the mechanisms underlying microbiome functions [52]. Moreover, SynComs are more effective in promoting plant growth and inhibiting disease severity than single species [52–54]. Here, a mixture of these three *Bacillus* strains (BcGE1, BaGE2, and BaGE3) was constructed as SynCom1. To investigate the potential of BcGE1, BaGE2, BaGE3, and SynCom1 in affecting plant growth, we first performed standard assays to detect their plant-beneficial functions, including bacterial siderophores production, phosphate solubilization, potassium solubilization, auxin secretion, and 1-aminocyclopropane-1-carboxylate (ACC) deaminase production. The results showed that BcGE1 and BcGE3 could produce auxin and ACC deaminase, and BcGE2 could produce siderophores, auxin, and ACC deaminase. SynCom1 showed more plant-beneficial traits than the individual strain, including the production of siderophores, auxin, and ACC deaminase and solubilization of phosphate. All the three *Bacillus* strains and SynCom1 could not solubilize potassium (Fig. S12). We next examine their antagonistic activity against *Aspergillus.* To this end, SynCom2, including *B. halotolerans* GE7, *Paenibacillus* sp. GE8, and *B. siamensis* GE10, was also set up as a control of SynCom1. Given that peanut pod diseases are often caused by multiple *Aspergillus* species concurrently in natural systems [55], a mixture containing *A. flavus* GE1 and *A. niger* GE2 was also included. An *ex situ* bacterial–fungal interaction screen was first developed to determine the effects of single strain and SynComs on *Aspergillus* growth [53]. Briefly, spores were collected from sporulating fungal isolates and were distributed into 96-well plates containing liquid tryptic soy broth (TSB) medium (20%) with or without a single strain or SynCom. Fungal growth was determined by fluorescence analysis with a wheat germ agglutinin (WGA), Alexa Fluor 488 conjugate. Regardless of whether *A. flavus* GE1 or *A. niger* GE2 single inoculation or coinoculation was performed, SynCom1 was better at inhibiting *Aspergillus* growth than single *Bacillus* strains and SynCom2 (Fig. 6A). We next inoculated *Bacillus* and *Aspergillus* isolates on pods and measured the abundance of *Aspergillus* to determine whether *Bacillus* can protect pods from *Aspergillus* infection (Fig. 6B). We found that SynCom1 further reduced the abundance of *A. flavus* GE1 and *A. niger* GE2 on the pod surface, compared to single *Bacillus* strains and SynCom2 (Fig. 6B–D). Moreover, when *Bacillus* and *Aspergillus* isolates were coinoculated on the fruits or tubers of other crops including maize, potato, apple, and strawberry, we found that SynCom1 was also better at reducing the abundance of *A. flavus* GE1 and *A. niger* GE2 on the surface of maize kernels, potato tubers, apple fruits, and strawberry fruits, compared to single *Bacillus* strain and SynCom2 (Fig. S13). These results further revealed that *Bacillus* consortia as protective microbiota function in reducing *Aspergillus* accumulation and suggested that the SynCom was better at inhibiting *Aspergillus* growth and reducing fruit disease than individual species.

### Transcriptome analyses of *A. flavus* and *A. niger* when coinoculation with SynCom1

**Figure 7:**
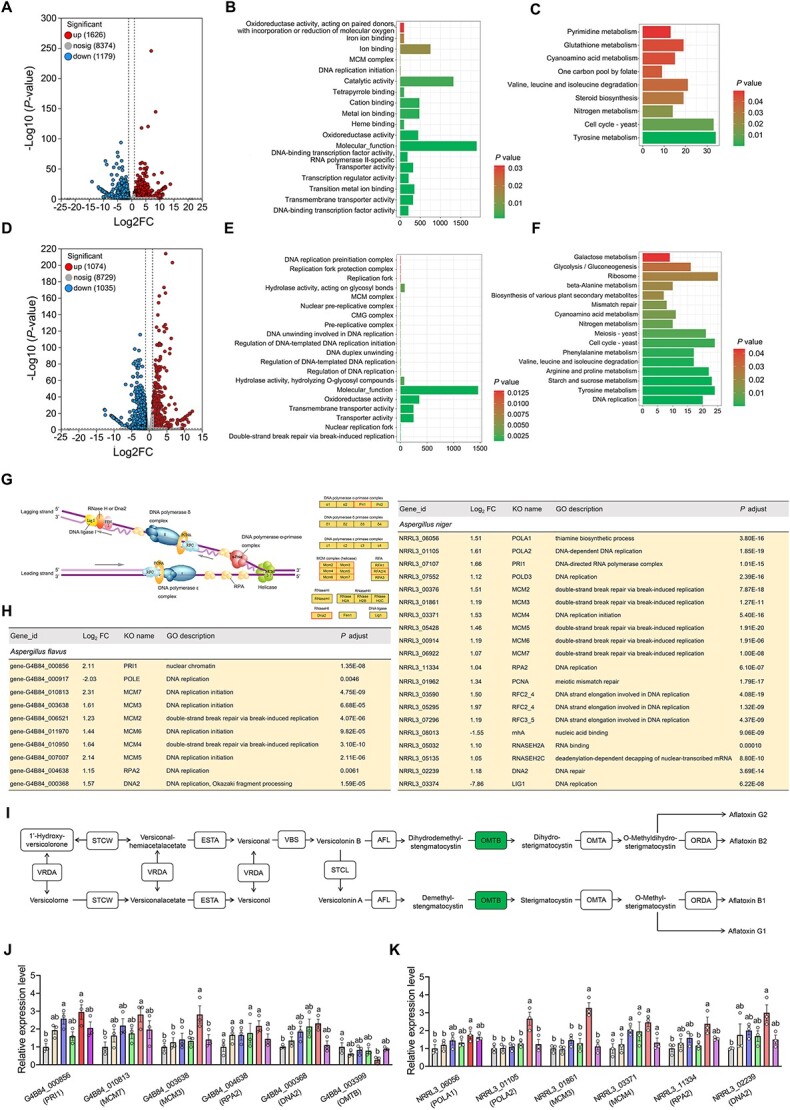
Transcriptome analyses of *A. flavus* and *A. niger* when cocultivation with SynCom1. (A, D) Volcano plots showing *A. flavus* (A) and *A. niger* DEGs (D) in single cultivation and SynCom1 cocultivation groups. (B, E) GO term analysis of DEGs from *A. flavus* vs. *A. flavus* + SynCom1 (B) and *A. niger* vs. *A. niger* + SynCom1 (E). (C, F) KEGG pathways analysis of DEGs from *A. flavus* vs. *A. flavus* + SynCom1 (C) and *A. niger* vs. *A. niger* + SynCom1 (F). (G) Image showing the KEGG-based depiction of DNA replication in eukaryotes. Functions supported by upregulated or nonregulated transcripts in *A. flavus* and *A. niger* are shown. (H) Tables showing the lists of transcripts related to DNA replication from *A. flavus* vs. *A. flavus* + SynCom1 and *A. niger* vs. *A. niger* + SynCom1. (I) The *omtB* in aflatoxin biosynthesis pathway in *A. flavus* was downregulated by SynCom1 following KEGG analysis. (J, K) qRT-PCR detection of 6 selected genes in *A. flavus* (J) and *A. niger* (K) in the presence of BcGE1, BaGE2, BaGE3, SynCom1, and SynCom2. Data are the mean ± SEM (*n* = 3 individual replicates). Different letters indicate significant differences among treatments (^*^P < 0.05, one-way analysis of variance followed by Tukey’s honest significant difference test). DEGs, differentially expressed genes; GO, Gene Ontology; KEGG, Kyoto Encyclopedia of Genes and Genomes; SynCom, synthetic microbial community.

RNA-seq was performed to reveal the molecular mechanisms by which SynCom1 inhibited the growth of *A. flavus* GE1 and *A. niger* GE2*.* Principal component analysis (PCA) and hierarchical cluster analyses of transcriptome data revealed a high similarity among the three biological replicates within each treatment (Figs S14 and S15). In *A. flavus*, 1626 genes were upregulated and 1179 genes were downregulated (fold change >2 or <0.05, *P* < 0.05) when coinoculated with SynCom1 (Fig. 7A; Table S8). In *A. niger*, the presence of SynCom1 resulted in 2109 DEGs, of which 1074 were upregulated and 1035 genes were downregulated (Fig. 7D; Table S9).

**Figure 8:**
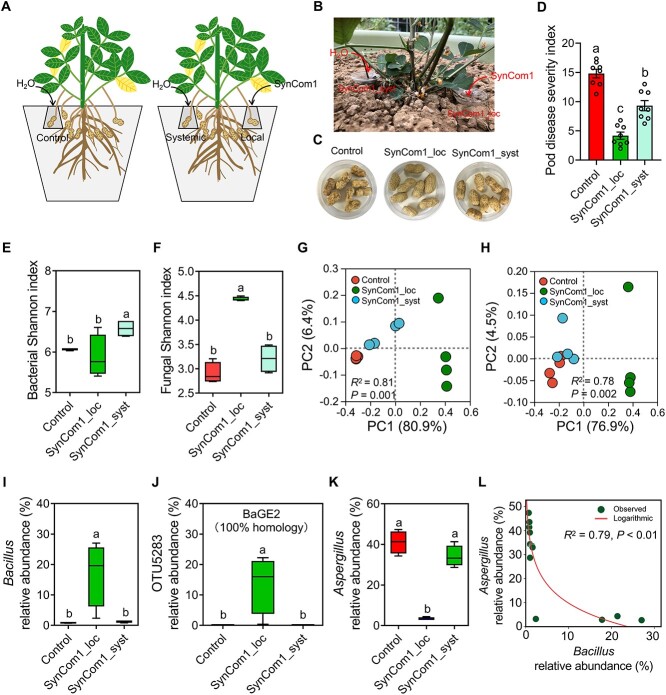
Reduction in pod diseases by SynCom1 with MP-conditioned soil cultivation. (A, B) Pot experiment 2 setup. Pot experiment 2 contained the following treatments: (i) control, Erlenmeyer flask with 1 ml of sterile water inoculation; (ii) Syncom1_local, Erlenmeyer flask with 1 ml of SynCom1 (BcGE1 + BaGE2 + BaGE3) suspension inoculation; and (iii) Syncom1_systemic, Erlenmeyer flask with 1 ml of sterile water inoculation. (C, D) SynCom1 treatment reduced the disease severity index of SynCom1_local and SynCom1_systemic pods compared to the control. Data are the mean ± SEM (*n* = 8 individual replicates). Different letters indicate significant differences among treatments (^*^P < 0.05, one-way analysis of variance followed by Tukey’s honest significant difference test). (E, F) Bacterial (E) and fungal (F) Shannon index in control, SynCom1_local, and SynCom1_systemic samples. Boxplots indicate median (middle line), 25th, 75th percentiles (box), and maximum and minimum values (whiskers) (*n* = 4 individual replicates). Different letters indicate significant differences among treatments (*P* < 0.05, one-way analysis of variance followed by Tukey’s honest significant difference test). (G) PCoA (based on the relative abundance of bacterial OTUs) of Bray–Curtis distances of control, SynCom1_local, and SynCom1_systemic samples. (H) PCoA (based on the relative abundance of fungal OTUs) of Bray–Curtis distances of control, SynCom1_local, and SynCom1_systemic samples. PERMANOVA was performed using the adonis function from the R package. (I–K) Relative abundance of *Bacillus* (I), OTU5283 (J), and *Aspergillus* (K) genus in control, SynCom1_local, and SynCom1_systemic samples. Boxplots indicate median (middle line), 25th, 75th percentiles (box), and maximum and minimum values (whiskers) (*n* = 4 individual replicates). Different letters indicate significant differences among treatments (^*^P < 0.05, one-way analysis of variance followed by Tukey’s honest significant difference test). (L) Correlation relationship between relative abundance of *Bacillus* and relative abundance of *Aspergillus*. SynCom, synthetic microbial community.

When determining GO terms that were significantly enriched (Benjamini–Hochberg false discovery rate–adjusted *P* value <0.05) of DEGs, we identified many biological process and cellular component terms that were associated with DNA replication and cell cycle, including DNA replication initiation (GO:0006270), MCM complex (GO:0042555), double-strand break repair via break-induced replication (GO:0000727), nuclear replication fork (GO:0043596), regulation of DNA replication (GO:0006275), regulation of DNA-templated DNA replication (GO:0090329), DNA duplex unwinding (GO:0032508), regulation of DNA-templated DNA replication initiation (GO:0030174), DNA unwinding involved in DNA replication (GO:0006268), pre-replicative complex (GO:0036387), CMG complex (GO:0071162), nuclear pre-replicative complex (GO:0005656), MCM complex (GO:0042555), replication fork (GO:0005657), replication fork protection complex (GO:0031298), and DNA replication preinitiation complex (GO:0031261) (Fig. 7B and E; Table S10). Consistently, KEGG pathway analysis of DEGs revealed that cell cycle-yeast (map04111) and DNA replication (map03030) were significantly enriched by the SynCom1 (Benjamini–Hochberg false discovery rate–adjusted *P* value <0.05) (Fig. 7C and F; Table S11). Contrary to our expectation, after screening for genes involved in eukaryotic DNA replication, the transcripts encoding the DNA polymerase ɑ-primase complex, MCM complex, RPA, and RNaseHI were upregulated in the presence of SynCom1 (Fig. 7G and H). This might be due to the different responses of proximate and distal hyphae to SynCom1. Nitroblue tetrazolium (NBT) staining found that reactive oxygen species (ROS) was obviously accumulated in the proximate hyphae rather than the distal hyphae (Fig. S16A), suggesting that proximate hyphae are subjected to stress from the SynCom1. Moreover, microscopic observation found that proximal hyphae were twisted and swelled, a typical characteristic of hyphae under stressful conditions. In contrast, distal hyphae were producing spores (Fig. S16B). In addition, a substantial number of transcripts encoding transmembrane transporters in *A. flavus* and *A. niger*, which are associated with transport of carbohydrates and amino acids, were regulated by SynCom1. For instance, transcripts encoding MFS general substrate transporters and ABC transporters were significantly regulated (Fig. 7B and E; Tables S8 and S9). Consistent with this, amino acid metabolism, including tyrosine metabolism, arginine and proline metabolism; valine, leucine, and isoleucine degradation; and glutathione metabolism, carbohydrate metabolism, including starch and sucrose metabolism, and galactose metabolism were enriched by SynCom1 (Fig. 7C and F). As aflatoxin is the main mycotoxin of *A. flavus*, the aflatoxin biosynthesis pathway in *A. flavus* was analyzed. A transcript (G4B84_003399) encoding O-methyltransferase B (OMTB) was downregulated in *A. flavus* in response to SynCom1 (Fig. 7I). These results suggest that the SynCom1 interferes fungal cell proliferation, metabolism, and mycotoxin biosynthesis in *Aspergillus.*

To reveal why SynCom1 was better at in inhibiting *Aspergillus* growth than the individual strain and SynCom2, six genes associated with the fungal cell cycle and mycotoxin biosynthesis from *A. flavus* and *A. niger,* respectively, were selected for qRT-PCR analysis. Compared to the single *Bacillus* strain and SynCom2, SynCom1 induced the largest changes in expression in three genes, including *MCM3* in *A. flavus* and *POLA2* and *MCM3* in *A. niger* (Fig. 7J and K)*.* MCM3 and POLA2 are associated with DNA replication and cell cycle (Fig. 7G).

### Reduction in pod disease by SynCom1 with monocropping-conditioned soil cultivation

In addition to pathogen growth inhibition, beneficial microbes can reduce disease incidence by activating ISR [16, 17]. We thus asked whether the SynCom1 has the ability to locally and systemically reduce pod diseases under MP conditions. Pot experiment 2 contained the following treatments: (i) control, Erlenmeyer flask with 1 ml of sterile water inoculation; (ii) Syncom1_local, Erlenmeyer flask with 1 ml of SynCom1 suspension inoculation; and (iii) Syncom1_systemic, Erlenmeyer flask with 1 ml of sterile water inoculation (Fig. 8A and B). We analyzed the disease severity of local and systemic pods at 30 days after SynCom1 inoculation. Compared to the control, treatment with SynCom1 reduced the disease severity in local and systemic pods by 56.78% and 32.51%, respectively (Fig. 8C and D). These data suggested that the SynCom1 treatment not only reduced the local disease severity in pods but also induced a systemic resistance to pathogens of pods within the same plants.

To reveal the mechanisms underlying the pod disease declines caused by the SynCom1, we analyzed the bacterial and fungal communities of GE samples in the control, SynCom1_local, and Syncom1_systemic treatments. Alpha-diversity analysis (based on Shannon and Chao1 indices) showed that SynCom1 inoculation increased the bacterial diversity in the Syncom1_systemic samples (Fig. 8E; Fig. S17; Table S12) and fungal diversity in the Syncom1_local samples (Fig. 8F; Fig. S17; Table S13). PERMANOVA confirmed the effect of Syncom1 on bacterial and fungal communities (bacteria: *R*^2^ = 0.81, *P* = .001; fungi: *R*^2^ = 0.77, *P* = .002) (Fig. 8G and H). A significant separation in the bacterial community between the control and SynCom1_systemic groups was found (*R*^2^ = 0.49, *P* = .027, Fig. S18A). In contrast, the Syncom1 inoculation did not significantly affect the fungal community between the control and SynCom1_systemic groups (*R*^2^ = 0.21, *P* = .11; Fig. S18B). When analyzing the bacterial community at the genus level, we found that treatment with SynCom1 significantly increased the relative abundance of *Bacillus* in SynCom1_local (Fig. 8I). By screening the *Bacillus* OTUs with SynCom1, we found that OTU11311, OTU5283, and OTU11225 shared 99.76%, 100%, and 100% homology with BcGE1, BaGE2, and BaGE3, respectively (Fig. 8J; Fig. S19). The increase in *Bacillus* relative abundance was mostly attributed to the OTU5283 (Fig. 8J). Compared to the control and SynCom1_systemic, SynCom1 significantly reduced *Aspergillus* relative abundance in SynCom1_local (Fig. 8K). Moreover, no significant difference in the relative abundance of *Bacillus* and *Aspergillus* genera was found between the control and Syncom1_systemic groups (Fig. 8I and K). A significant negative correlation was found between *Bacillus* and *Aspergillus* abundance in the present pot experiment (Fig. 8L). Moreover, OTU5283 abundance was also negatively correlated with *Aspergillus* abundance (Fig. S20).

### Activation of ISR by SynCom1

**Figure 9:**
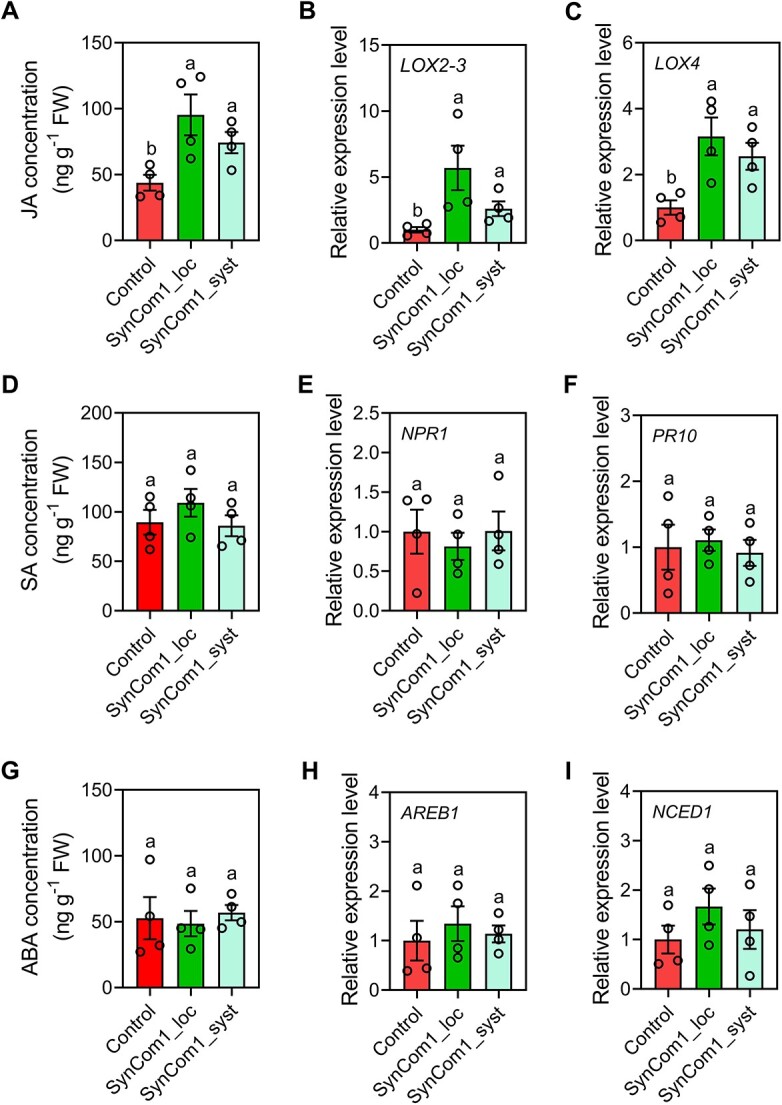
Activation of ISR by SynCom1. (A, D, G) JA (A), SA (D), and ABA (G) levels in the shells of control, local, and systemic pods of peanut plants treated with SynCom1. Data are the mean ± SEM (*n* = 4 individual replicates). Different letters indicate significant differences among treatments (*P* < 0.05, one-way analysis of variance followed by Tukey’s honest significant difference test). (B, C, E, F, H, I) Relative expression levels of JA (B, C), SA (E, F), and ABA (H, I) signaling marker genes in the shells of control, local, and systemic pods of peanut plants treated with SynCom1. The data are the mean ± SEM (*n* = 4 individual replicates). Different letters indicate significant differences among treatments (^*^P < 0.05, one-way analysis of variance followed by Tukey’s honest significant difference test). JA, jasmonic acid; SA, salicylic acid; ABA, abscisic acid. *LOX2–3*, *lipoxygenase 2–3*; *LOX4*, *lipoxygenase 4*; *NPR1*, *Nonexpressor of pathogenesis-related gene 1*; *PR10*, *pathogenesis-related class 10 protein*; *AREB1*, *ABA-responsive element 1*; *NECD1*, *9-cis-epoxycarotenoid dioxygenase*; SynCom, synthetic microbial community.

Because there was no significant difference in the relative abundance of *Bacillus* and *Aspergillus* genera in the control and Syncom1_systemic GE samples (Fig. 8I and K), we hypothesized that the SynCom1 treatment reduced pod disease in Syncom1_systemic by activating the ISR. We tested this hypothesis by detecting the contents of JA, SA, and ABA and analyzing the expression patterns of the defense-related marker genes associated with JA, SA, and ABA in the pod shells. Compared with the control, treatment with SynCom1 increased the JA content in pod shells of Syncom1_local and Syncom1_systemic by 2.18- and 1.70-fold, respectively, at 30 days after inoculation (Fig. 9A). Moreover, compared with the control, SynCom1 upregulated the expression of the JA signaling marker genes *LOX2–3* and *LOX4* in local and systemic pod shells by 4.69- and 2.60-fold, respectively (Fig. 9B and C). However, the levels of SA and ABA and the expression of SA and ABA signaling marker genes in pod shells of control, Syncom1_local, and Syncom1_systemic treatments were not significantly altered by SynCom1 inoculation (Fig. 9D–I). These data show that the SynCom1 inoculation reduced pod disease by not only inhibiting *Aspergillus* growth but also priming JA-mediated ISR in pod shells.

## Discussion

Plant-associated microbiomes play important roles in plant health and productivity [1, 2, 56, 57], whereas this knowledge has mostly been obtained from the studies of roots and leaves and their associated microbial communities. However, unlike roots and leaves, which are present throughout a large part of the plant life cycle [7], fruits, as reproductive organs, develop on mature plants and are often present for a limited period. Thus, whether the knowledge gained from vegetative organs applied to plant reproductive organs is still unknown. Some plants, such as peanut, develop fruits underground, and these fruits share the same growing environment with roots [58, 59]. However, little is known about the differences in the microbial features of underground fruits and roots and potential links between the microbiome and fruit health. Here, we profiled the microbiome from the bulk, rhizosphere, and geocarposphere soils of peanut under monocropping and rotation management regimes and revealed the links geocarposphere microbiome and pod disease. Our study revealed that the plant underground fruits harbored a different microbiome from roots and that the depletion of key protective bacterial communities promoted the incidence of fruit disease.

In the present study, cropping regimes had a significant effect on microbiome composition. Cropping regimes influence the plant-associated microbiomes by altering the initial soil microbiome, plant physiology, and phenotype [58–60]. A previous study reported that monocropping resulted in root disease outbreaks by increasing the abundance of fungal pathogens in the rhizosphere [60]. Consistent with this, our results found that the abundance of *Fusarium* was higher in the rhizosphere under monocropping than that under rotation. *Fusarium* species are the major fungal pathogens causing root rot of many crops [60, 61]. Similarly, the occurrence of pod disease increased under monocropping conditions. Geocarposphere microbiome analysis, combined with *in vitro* and *in vivo Aspergillus* infection experiments, we confirmed that *Aspergillus* accumulation is the cause of pod disease under monocropping conditions. *Aspergillus* species, including *A. flavus* and *A. niger*, as ubiquitous fungal saprophytes, are the causal agent of disease in many kinds of fruits, including peanut pods [62, 63]. In the peanut monocropping system, *Aspergillus* remains in the soil and litter after harvest, which could cause disease in the pods of the next generation, also known as negative plant–soil feedback. In addition, *Aspergillus* on fruits can produce mycotoxins that threaten human health. For example, *A. parasiticus*-derived aflatoxins B1, B2, G1, and G2 are detrimental to the human liver, epididymis, testis, kidney, and heart [64].

Regardless of monocropping or rotation conditions, clear differences in bacterial and fungal communities were found between the rhizosphere and geocarposphere, suggesting that plant organs filter their surrounding microbial communities [65]. This raised a fundamental question: what are the driving forces that cause the differences in microbial communities between the rhizosphere and geocarposphere? We propose two explanations for these differences. First, it is possible that the roots and pods harbored different exudate compositions. It has been established that root exudates, such as amino acids, organic acids, flavonoids, and alkaloids, drive rhizosphere microbial community assembly [66, 67]. For example, coumarins, and benzoxazinoids exuded by roots play a role in shaping the rhizosphere microbiome [68–70]. By comparison, little is known about the exudate composition of pods. Further study with liquid chromatography–mass spectrometry is required to dissect the differences in the compositions of root and pod exudates. The second possible scenario underlying microbial differences between rhizosphere and geocarposphere is the structures of roots and pods. Compared to plant chemistry, the roles of plant organ structure in sharping the microbial community are underrated. For example, a high CK tomato genotype (*pBLS >> IPT*) exhibited altered structural features of the leaf surface, such as smaller epidermal and mesophyll cells, and increased amounts of nonglandular trichomes, which could support colonization of Gram-positive bacilli [71]. As the root system includes different types of primary (stem-attached large roots), secondary (lateral roots), and tertiary (hairy-like fine) roots [72], the structural heterogeneities of roots are higher than those of pods. As a consequence, roots can create more ecological niches on the surface and in the rhizosphere for diverse microbial species. Consistent with this, the bacterial Shannon index in RP-YR samples was higher than that in RP-GE samples.

The eubiosis of the microbial community is key to plant biotic and abiotic stress tolerance, which sustains plant health [3, 4, 73]. Compared to the effects of rotation, the features of bacterial and fungal communities in the geocarposphere at the genus level mostly strongly influenced by monocropping were the increased *Aspergillus* abundance and decreased *Bacillus* abundance. The changes in *Bacillus* abundance were due to the cropping regimes, as MP reduced the abundance of *Bacillus* in the BS, YR, OR, and GE samples compared to RP. In comparison, *Aspergillus* was specifically accumulated in MP-GE, suggesting that the geocarposphere environment created by pods under MP conditions favors *Aspergillus*. Notably, reduced *Bacillus* but similar *Aspergillus* abundances were found in the rhizosphere under MP conditions, compared to RP conditions. This may be because *Aspergillus* species prefer to infect fruits rather than roots [63, 64], or factors other than *Bacillus* may be involved in restricting *Aspergillus* accumulation in the rhizosphere. Moreover, the genera *Sphingomonas*, *Streptomyces*, and *Bradyrhizobium*, which commonly exhibit antifungal activity and plant growth promotion, were enriched in MP-GE. This could be due to the emergency and development of disease-suppressive soils during long-term monocropping. Long-term monocropping results in accumulation of soil pathogens and outbreaks of plant diseases via a phenomenon known as negative plant–soil feedback. During this process, disease-suppressive bacteria will appear and accumulate in the soils after monocropping for several years [74, 75].

Pairwise correlation analyses among pod disease, *Aspergillus* abundance, and *Bacillus* abundance, combined with pod coinoculation with *Aspergillus* and *Bacillus* experiments, suggested that the imbalance in *Aspergillus* and *Bacillus* in geocarposphere resulted in pod disease. The genus *Bacillus* is one of most exploited microbial groups that inhibit the growth of fungal pathogens by producing antimicrobials [76]. Moreover, *Bacillus* species often act as key microbial community members that maintain plant health via direct antagonistic effects and/or by inducing plant immunity [77]. Consistent with this, three *Bacillus* species isolated from the geocarposphere under rotation with high culturable abundance showed direct antagonism against *A. flavus* and *A. niger*. Moreover, a SynCom1 containing the three *Bacillus* isolates displayed higher antagonistic activity against *A. flavus* and *A. niger* than its constituent individual strains. This is consistent with other studies showing that mixed microbial consortia excelled in inhibiting pathogen growth and reducing plant diseases [25, 45]. The better antagonistic activities against *A. flavus* and *A. niger* by SynCom1 were also confirmed by the transcriptome and qRT-PCR data, as SynCom1 exhibited the largest changes in expression of genes associated with fungal cell proliferation and aflatoxin biosynthesis than single strain. The increased expression of genes related to fungal cell proliferation in the cocultivation system might be due to the increased spores production of hyphae distal to the SynCom1 stream, as the proximal hyphae were damaged by SynCom1. This indicates an adaptive strategy by *A. flavus* and *A. niger* during cocultivation with SynCom1. The fast growth and mismatch repair of fungi could promote their survival under a stressful environment [78, 79]. Moreover, enrichment of mismatch repair pathway and abnormal morphology of hyphae indicated that antimicrobials production is involved in SynCom1-mediated *Aspergillus* growth inhibition [80]. The *omtB*, encoding OMTB, which is responsible for the conversion of demethylsterigmatocystin (DMST) and dihydrodemethylsterigmatocystin (DHDMST) to sterigmatocystin (ST) and dihydrosterigmatocystin (DHST) in aflatoxin biosynthesis pathway [81], was downregulated in the cocultivation system, indicating that the SynCom1 impairs the mycotoxins biosynthesis in the *Aspergillus*. The amplified antagonistic activity exhibited by the SynCom may be attributed to interactions or functional complementarity among three *Bacillus* isolates. This was indirectly supported by the results that the SynCom1 exhibited more plant-beneficial traits than the individual strain. Introduction of microbial consortia can increase their survival and ecosystem functions in soil conditions through metabolic interactions [82]. *Bacillus* consortia can increase the amount and diversity of antimicrobials, as different *Bacillus* species can produce different kinds of antimicrobials [83]. Mixed antimicrobials are more effective in antagonism against pathogens than single antimicrobials [84]. Taken together, these data provided evidence that the depletion of *Bacillus* resulted in the accumulation of *Aspergillus* in the geocarposphere and thus promoted the incidence of pod disease.

Treatment with the SynCom1 increased *Bacillus* abundance and reduced *Aspergillus* abundance in geocarposphere and pod disease occurrence under monocropping conditions, further supporting the direct antagonistic activity exerted against *Aspergillus* by SynCom1. The increased *Bacillus* abundance in SynCom1_local was largely attributed to *B. amyloliquefaciens* GE2 (OTU5283). However, little is known about why BaGE2 is better at geocarposphere colonization than BcGE1 and BaGE3. This process might be associated with the microbial survival strategy and interactions between the introduced SynCom1 and the preexisting microbial community in the soil [27, 77]. The SynCom1 reduced the disease severity of adjacent pods within the same plant; however, the levels of *Aspergillus* abundance were similar in the geocarposphere of adjacent and control pods. Plant disease severity is determined by the abundance of pathogens, the level of plant immunity, and the environment [85]. By screening plant phytohormones and their signaling gene expression, we confirmed that JA-mediated systemic resistance was induced by the SynCom1. The systemic effect of SynCom1 on pods was also supported by the bacterial community analysis, in which SynCom1 altered the bacterial ɑ and β diversity of systemic GE samples compared with the control. Similarly, a SynCom with four *Bacillus* species activated JA signaling-dependent ISR against *Ralstonia solanacearum* in tomato [3]. As soil is heterogeneous in microbial community composition and beneficial microbes are unlikely to be evenly distributed around plant underground organs, the presence of ISR is vital to plant health. Beneficial microbes activate ISR in a manner dependent on microbe-associated molecular patterns (MAMPs), such as flagellin, elongation factor Tu (EF-Tu), lipopolysaccharide (LPS), and lipopeptides [16, 86, 87]. Thus, the SynCom1 in the present study may contain a cocktail of MAMPs, as *Bacillus* species harbored different MAMPs. *B. subtilis*, for instance, produces cyclic lipopeptides, including surfactins, mycosubtilin, and plipastatin, which activate SA and JA signaling pathways [88]. Intriguingly, surfactins, mycosubtilin, and plipastatin displayed direct antifungal activity [89, 90], suggesting the multifunctionality of microbial MAMPs. Although a single *Bacillus* strain has been known to elicit ISR, the SynCom-derived molecular determinants are likely complex and should be investigated further.

## Conclusion

In this study, using peanut as a research material, we revealed the microbiome specificity of plant organs and that depletion of the key bacterial community promotes the incidence of fruit disease. Although roots and pods of peanut share the same growth environment, their surrounding microbial communities are different, indicating the filter effect of plant organs on the microbiome. Moreover, at the genus level, the most common microbial feature in the geocarposphere under monocropping and rotation conditions is the imbalance of *Aspergillus* and *Bacillus*. By investigating the interactions between the *Aspergillus* and *Bacillus* strains, geocarposphere microbiome analysis, and plant immunity analysis, the outbreak of pod disease under monocropping conditions was found to be due to the depletion of protective *Bacillus* consortia in the geocarposphere. Although the conclusions in the present study cannot extend to all other crops, for instance, tree crops, which fruit aboveground, but our study highlights the importance of microbiome in fruit health. Moreover, our study supports the application of synthetic microbial consortia in controlling *Aspergillus*-derived fruit disease. Further investigations are needed to (i) identify the driving forces responsible for the filter effect of roots and pods on microbiome, (ii) reveal the mechanisms underlying the colonization advantage of BaGE2 in the geocarposphere following the introduction of SynCom, and (iii) understand the determinants of SynCom that suppress pathogens and activate plant immunity.

## Supplementary Material

Supplementary_Information_wrae071

Supplementary_Tables_wrae071

## Data Availability

The raw sequencing data of microbiota has been deposited under BioProject PRJNA989386, PRJNA992791, PRJNA989419, and PRJNA989426. The accession numbers for the RNA sequencing data are PRJNA989718 and PRJNA989723. All data supporting the findings of this study are available in the manuscript or supplementary information.

## References

[1] Trivedi P , Leach JE, Tringe SG et al. Plant-microbiome interactions: from community assembly to plant health. Nat Rev Microbiol 2020;18:607–21. 32788714 10.1038/s41579-020-0412-1

[2] de Vries FT , Griffiths RI, Knight CG et al. Harnessing rhizosphere microbiomes for drought-resilient crop production. Science 2020;368:270–4. 32299947 10.1126/science.aaz5192

[3] Lee SM , Kong HG, Song GC et al. Disruption of Firmicutes and Actinobacteria abundance in tomato rhizosphere causes the incidence of bacterial wilt disease. ISME J 2021;15:330–47. 33028974 10.1038/s41396-020-00785-x PMC7852523

[4] Chen T , Nomura K, Wang X et al. A plant genetic network for preventing dysbiosis in the phyllosphere. Nature 2020;580:653–7. 32350464 10.1038/s41586-020-2185-0 PMC7197412

[5] Armault G , Mony C, Vandenkoornhuyse P. Plant microbiota dysbiosis and the Anna Karenina principle. Trends Plant Sci 2023;28:18–30. 36127241 10.1016/j.tplants.2022.08.012

[6] Jing J , Cong WF, Martijin BT. Legacies at work: plant-soil-microbiome interactions underpinning agricultural sustainability. Trends Plant Sci 2022;27:781–92. 35701291 10.1016/j.tplants.2022.05.007

[7] Cui Z , Huntley RB, Zeng Q et al. Temporal and spatial dynamics in the apple flower microbiome in the presence of the phytopathogen *Erwinia amylovora*. ISME J 2021;15:318–29. 33024293 10.1038/s41396-020-00784-y PMC7853089

[8] Nelson EB . The seed microbiome: origins, interactions, and impacts. Plant Soil 2018;422:7–34.

[9] Simonin M , Briand M, Chesneau G et al. Seed microbiota revealed by a large-scale meta-analysis including 50 plant species. New Phytol 2022;234:1448–63. 35175621 10.1111/nph.18037

[10] Luo CX , Schnabel G, Hu M et al. Global distribution and management of peach diseases. Phytopathol Res 2022;4:30.

[11] Tian Y , Li E, Liang Z et al. Diagnosis of typical apple diseases: a deep learning method based on multi-scale dense classification network. Front Plant Sci 2021;12:698474. 34659279 10.3389/fpls.2021.698474 PMC8517256

[12] Xu Y , Tong Z, Zhang X et al. Plant volatile organic compound (E)-2-hexenal facilitates *Botrytis cinerea* infection of fruits by inducing sulfate assimilation. New Phytol 2021;231:432–46. 33792940 10.1111/nph.17378

[13] Nayak SN , Agarwal G, Pandey MK et al. *Aspergillus flavus* infection triggered immune responses and host-pathogen cross-talks in groundnut during *in-vitro* seed colonization. Sci Rep 2017;7:9659. 28851929 10.1038/s41598-017-09260-8 PMC5574979

[14] Zhu GY , Wang X, Chen TM et al. First report of *aspergillus flavus* causing fruit rot on kiwifruit in China. Plant Dis 2022;160:1990. 10.1094/PDIS-08-21-1771-PDN

[15] Palmer MG , Mansouripour SM, Blauer KA et al. First report of *aspergillus tubingensis* causing strawberry fruit rot in California. Plant Dis 2019;103:2948–9.

[16] Pieterse CMJ , Zamioudis C, Berendsen RL et al. Induced systemic resistance by beneficial microbes. Annu Rev Phytopathol 2014;52:347–75. 24906124 10.1146/annurev-phyto-082712-102340

[17] Liu H , Li J, Carvalhais LC et al. Evidence for the plant recruitment of beneficial microbes to suppress soil-borne pathogens. New Phytol 2021;229:2873–85. 33131088 10.1111/nph.17057

[18] Raaijmakers JM , Weller DM. Natural plant protection by 2,4-diacetylphloroglucinol-producing *Pseudomonas* spp. in take-all decline soils. Mol Plant-Microbe Interact 1998;11:144–52.

[19] Pieterse CMJ , Berendsen RL, de Jonge R et al. *Pseudomonas simiae* WCS417: star track of a model beneficial rhizobacterium. Plant Soil 2021;461:245–63.

[20] Liu H , Brettell LE, Qiu Z et al. Microbiome-mediated stress resistance in plants. Trends Plant Sci 2020;25:733–43. 32345569 10.1016/j.tplants.2020.03.014

[21] Wang Z , Hu X, Solanki MK et al. A synthetic microbial community of plant core microbiome can be a potential biocontrol tool. J Agric Food Chem 2023;71:5030–41. 36946724 10.1021/acs.jafc.2c08017

[22] Karkaria B , Fedorec AJH, Barnes CP. Automated design of synthetic microbial communities. Nat Commun 2021;12:672. 33510148 10.1038/s41467-020-20756-2 PMC7844305

[23] Schmitz L , Yan Z, Schneijderberg M et al. Synthetic bacterial community derived from a desert rhizosphere confers salt stress resilience to tomato in the presence of a soil microbiome. ISME J 2022;16:1907–20. 35444261 10.1038/s41396-022-01238-3 PMC9296610

[24] Wang C , Li Y, Li M et al. Functional assembly of root-associated microbial consortia improves nutrient efficiency and yield in soybean. J Integr Plant Biol 2021;63:1021–35. 33491865 10.1111/jipb.13073

[25] Niu B , Paulson JN, Zheng X et al. Simplified and representative bacterial community of maize roots. Proc Natl Acad Sci USA 2017;114:E2450–9. 28275097 10.1073/pnas.1616148114 PMC5373366

[26] Wei N , Ashman TL. The effects of host species and sexual dimorphism differ among root, leaf, and flower microbiomes of wild strawberries *in situ*. Sci Rep 2018;8:5195. 29581521 10.1038/s41598-018-23518-9 PMC5979953

[27] Liu YX , Qin Y, Bai Y. Reductionist synthetic community approaches in root microbiome research. Curr Opin Microbiol 2019;49:97–102. 31734603 10.1016/j.mib.2019.10.010

[28] Massoni J , Bortfeld-Miller M, Widmer A et al. Capacity of soil bacteria to reach the phyllosphere and convergence of floral communities despite soil microbiota variation. Proc Natl Acad Sci USA 2021;118:e2100150118. 34620708 10.1073/pnas.2100150118 PMC8521660

[29] Vorholt JA . Microbial life in the phyllosphere. Nat Rev Microbiol 2012;10:828–40. 23154261 10.1038/nrmicro2910

[30] Humphrey PT , Whiteman NK. Insect herbivory reshapes a native leaf microbiome. Nat Ecol Evol 2020;4:221–9. 31988447 10.1038/s41559-019-1085-x PMC7332206

[31] Bai B , Liu W, Qiu X et al. The root microbiome: community assembly and its contributions to plant fitness. J Integr Plant Biol 2022;64:230–43. 35029016 10.1111/jipb.13226

[32] Chen X , Yang Q, Li H et al. Transcriptome-wide sequencing provides insights into geocarpy in peanut (*Arachis hypogaea* L.). Plant Biotechnol J 2016;14:1215–24. 26502832 10.1111/pbi.12487 PMC11388922

[33] Liu H , Liang X, Lu Q et al. Global transcriptome analysis of subterranean pod and seed in peanut (*Arachis hypogaea* L.) unravels the complexity of fruit development under dark condition. Sci Rep 2020;10:13050. 32747681 10.1038/s41598-020-69943-7 PMC7398922

[34] Shi W , Li M, Wei G et al. The occurrence of potato common scab correlates with the community composition and function of the geocaulosphere soil microbiome. Microbiome 2019;7:14. 30709420 10.1186/s40168-019-0629-2 PMC6359780

[35] Xu N , Tan G, Wang H et al. Effect of biochar additions to soil on nitrogen leaching, microbial biomass and bacterial community structure. Eur J Soil Biol 2016;74:1–8.

[36] Adams RI , Miletto M, Taylor JW et al. Dispersal in microbes: fungi in indoor air are dominated by outdoor air and show dispersal limitation at short distances. ISME J 2013;7:1262–73. 23426013 10.1038/ismej.2013.28 PMC3695294

[37] Caporaso JG , Kuczynski J, Stombaugh J et al. QIIME allows analysis of high-throughput community sequencing data. Nat Methods 2010;7:335–6. 20383131 10.1038/nmeth.f.303 PMC3156573

[38] Magoc T , Salzberg SL. FLASH: fast length adjustment of short reads to improve genome assemblies. Bioinformatics 2011;27:2957–63. 21903629 10.1093/bioinformatics/btr507 PMC3198573

[39] Edgar RC . UPARSE: highly accurate OTU sequences from microbial amplicon reads. Nat Methods 2013;10:996–8. 23955772 10.1038/nmeth.2604

[40] Oksanen J , Blanchet FG, Friendly M et al. vegan: Community ecology package. R package 2.5-6. https://CRAN.R-project.org/package=vegan (Accessed 1 September 2019).

[41] Segata N , Izard J, Waldron L et al. Metagenomic biomarker discovery and explanation. Genome Biol 2011;12:R60. 21702898 10.1186/gb-2011-12-6-r60 PMC3218848

[42] Xu F , Liao H, Zhang Y et al. Coordination of root auxin with the fungus *Piriformospora indica* and bacterium *Bacillus cereus* enhances rice rhizosheath formation under soil drying. ISME J 2022;16:801–11. 34621017 10.1038/s41396-021-01133-3 PMC8857228

[43] Santhanam R , Luu VT, Weinhold A et al. Native root-associated bacteria rescue a plant from a sudden-wilt disease that emerged during continuous cropping. Proc Natl Acad Sci USA 2015;112:E5013–20. 26305938 10.1073/pnas.1505765112 PMC4568709

[44] Gera Hol WH , Garbeva P, Hordijk C et al. Non-random species loss in bacterial communities reduces antifungal volatile production. Ecology 2015;96:2042–8. 26405729 10.1890/14-2359.1

[45] Zhou X , Wang J, Liu F et al. Cross-kingdom synthetic microbiota supports tomato suppression of Fusarium wilt disease. Nat Commun 2022;13:7890. 36550095 10.1038/s41467-022-35452-6 PMC9780251

[46] Al-Shuhaib MBS , Albakri AH, Alwan SH et al. Optimal PCR primers for rapid and accurate detection of *aspergillus flavus* isolates. Microb Pathog 2018;116:351–5. 29427712 10.1016/j.micpath.2018.01.049

[47] Peterson SW . Phylogenetic analysis of *aspergillus* species using DNA sequences from four loci. Mycologia 2008;100:205–26. 18595197 10.3852/mycologia.100.2.205

[48] Zhang W , Yuan J, Cheng T et al. Flowering-mediated root-fungus symbiosis loss is related to jasmonate-dependent root soluble sugar deprivation. Plant Cell Environ 2019;42:3208–26. 31373013 10.1111/pce.13636

[49] Chen P , He W, Shen Y et al. Interspecific neighbor stimulates peanut growth through modulating root endophytic microbial community construction. Front Plant Sci 2022;13:830666. 35310651 10.3389/fpls.2022.830666 PMC8928431

[50] Zhang W , Luo X, Mei YZ et al. Priming of rhizobial nodulation signaling in the mycosphere accelerates nodulation of legume hosts. New Phytol 2022;235:1212–30. 35488499 10.1111/nph.18192

[51] Li M , Pommier T, Yin Y et al. Indirect reduction of *Ralstonia solanacearum* via pathogen helper inhibition. ISME J 2022;16:868–75. 34671104 10.1038/s41396-021-01126-2 PMC8857195

[52] Compant S , Samad A, Faist H et al. A review on the plant microbiome: ecology, functions, and emerging trends in microbial application. J Adv Res 2019;19:29–37. 31341667 10.1016/j.jare.2019.03.004 PMC6630030

[53] Durán P , Thiergart T, Garrido-Oter R et al. Microbial interkingdom interactions in roots promote *Arabidopsis* survival. Cell 2018;175:973–83. 30388454 10.1016/j.cell.2018.10.020 PMC6218654

[54] Hou S , Thiergart T, Vannier N et al. A microbiota–root–shoot circuit favours *Arabidopsis* growth over defence under suboptimal light. Nat Plants 2021;7:1078–92. 34226690 10.1038/s41477-021-00956-4 PMC8367822

[55] von Hertwig AM , Iamanaka BT, Neto DPA et al. Interaction of *aspergillus flavus* and *A. Parasiticus* with *Salmonella* spp. isolated from peanuts. Int J Food Microbiol 2020;328:108666. 32454365 10.1016/j.ijfoodmicro.2020.108666

[56] Trivedi P , Mattupalli C, Eversole K et al. Enabling sustainable agriculture through understanding and enhancement of microbiomes. New Phytol 2021;230:2129–22147. 33657660 10.1111/nph.17319

[57] Morales Moreira ZP , Chen MY, Yanez Ortuno DL et al. Engineering plant microbiomes by integrating eco-evolutionary principles into current strategies. Curr Opin Plant Biol 2023;71:102316. 36442442 10.1016/j.pbi.2022.102316

[58] Li X , Jousset A, de Boer W et al. Legacy of land use history determines reprogramming of plant physiology by soil microbiome. ISME J 2019;13:738–51. 30368524 10.1038/s41396-018-0300-0 PMC6461838

[59] Li P , Liu J, Saleem M et al. Reduced chemodiversity suppresses rhizosphere microbiome functioning in the mono-cropped agroecosystems. Microbiome 2022;10:108. 35841078 10.1186/s40168-022-01287-y PMC9287909

[60] Li X , Ding C, Zhang T et al. Fungal pathogen accumulation at the expense of plant-beneficial fungi as a consequence of consecutive peanut monoculturing. Soil Biol Biochem 2014;72:11–8.

[61] Li X , de Boer W, Zhang Y et al. Suppression of soil-borne *Fusarium* pathogens of peanut by intercropping with the medicinal herb *Atractylodes lancea*. Soil Biol Biochem 2018;116:120–30.

[62] Xiao W , Yan Ps W, Hq LF. Antagonizing *aspergillus parasiticus* and promoting peanut growth of *Bacillus* isolated from peanut geocarposphere soil. J Integr Agric 2014;13:2445–51.

[63] Majumdar R , Lebar M, Mack B et al. The *aspergillus flavus* spermidine synthase (*spds*) gene, is required for normal development, aflatoxin production, and pathogenesis during infection of maize kernels. Front Plant Sci 2018;9:317. 29616053 10.3389/fpls.2018.00317 PMC5870473

[64] Kumar A , Pathak H, Bhadauria S et al. Aflatoxin contamination in food crops: causes, detection, and management: a review. Food Prod Process and Nutr 2021;3:17.

[65] Santoyo G . How plants recruit their microbiome? New insights into beneficial interactions. J Adv Res 2022;40:45–58. 36100333 10.1016/j.jare.2021.11.020 PMC9481936

[66] Sasse J , Martinoia E, Northen T. Feed your friends: do plant exudates shape the root microbiome? Trends Plant Sci 2018;23:25–41. 29050989 10.1016/j.tplants.2017.09.003

[67] Zhalnina K , Louie KB, Hao Z et al. Dynamic root exudate chemistry and microbial substrate preferences drive patterns in rhizosphere microbial community assembly. Nat Microbiol 2018;3:470–80. 29556109 10.1038/s41564-018-0129-3

[68] Stringlis IA , Yu K, Feussner K et al. MYB72-dependent coumarin exudation shapes root microbiome assembly to promote plant health. Proc Natl Acad Sci USA 2018;115:E5213–22. 29686086 10.1073/pnas.1722335115 PMC5984513

[69] Kudjordjie EN , Sapkota R, Steffensen SK et al. Maize synthesized benzoxazinoids affect the host associated microbiome. Microbiome 2019;7:59. 30975184 10.1186/s40168-019-0677-7 PMC6460791

[70] Hu L , Robert CAM et al. Root exudate metabolites drive plant-soil feedbacks on growth and defense by shaping the rhizosphere microbiota. Nat Commun 2018;9:2738. 30013066 10.1038/s41467-018-05122-7 PMC6048113

[71] Gupta R , Elkabetz D, Leibman-Markus M et al. Cytokinin drives assembly of the phyllosphere microbiome and promotes disease resistance through structural and chemical cues. ISME J 2022;16:122–37. 34272494 10.1038/s41396-021-01060-3 PMC8692462

[72] Pervaiz ZH , Contreras J, Hupp BM et al. Root microbiome changes with root branching order and root chemistry in peach rhizosphere soil. Rhizosphere 2020;16:100249.

[73] Wei Z , Gu Y, Friman VP et al. Initial soil microbiome composition and functioning predetermine future plant health. Sci Adv 2019;5:eaaw0759. 31579818 10.1126/sciadv.aaw0759 PMC6760924

[74] Weller DM , Raaijmakers JM, Gardener BB et al. Microbial populations responsible for specific soil suppressiveness to plant pathogens. Annu Rev Phytopathol 2002;40:309–48. 12147763 10.1146/annurev.phyto.40.030402.110010

[75] Berendsen RL , Pieterse CMJ, Bakker PAHM. The rhizosphere microbiome and plant health. Trends Plant Sci 2012;17:478–86. 22564542 10.1016/j.tplants.2012.04.001

[76] Saxena AK , Kumar M, Chakdar H et al. *Bacillus* species in soil as a natural resource for plant health and nutrition. J Appl Microbiol 2019;128:1583–94. 31705597 10.1111/jam.14506

[77] Zhang N , Wang Z, Shao J et al. Biocontrol mechanisms of *Bacillus*: improving the efficiency of green agriculture. Microb Biotechnol 2023;16:2250–63. 37837627 10.1111/1751-7915.14348 PMC10686189

[78] Fisher MC , Alastruey-Izquierdo A, Berman J et al. Tackling the emerging threat of antifungal resistance to human health. Nat Rev Microbiol 2022;20:557–71. 35352028 10.1038/s41579-022-00720-1 PMC8962932

[79] Billnyre RB , Clancey SA, Heitman J. Natural mismatch repair mutations mediate phenotypic diversity and drug resistance in *Cryptococcus deuterogattii*. elife 2017;6:e28802. 28948913 10.7554/eLife.28802 PMC5614558

[80] Poppeliers SWM , Sánchez-Gil JJ, de Jonge R. Microbes to support plant health: understanding bioinoculant success in complex conditions. Curr Opin Microbiol 2023;73:102286. 36878082 10.1016/j.mib.2023.102286

[81] Yu J , Woloshuk CP, Bhatnagar D et al. Cloning and characterization of *avfA* and *omtB* genes involved in aflatoxin biosynthesis in three *aspergillus* species. Gene 2020;248:157–67. 10.1016/s0378-1119(00)00126-8 10806361

[82] Sun X , Xu Z, Xie J et al. *Bacillus velezensis* stimulates resident rhizosphere *Pseudomonas stutzeri* for plant health through metabolic interactions. ISME J 2022;16:774–87. 34593997 10.1038/s41396-021-01125-3 PMC8483172

[83] Caukier S , Nannan C, Gillis A et al. Overview of the antimicrobial compounds produced by members of the *Bacillus subtilis* group. Front Microbiol 2019;10:302. 30873135 10.3389/fmicb.2019.00302 PMC6401651

[84] Rezzoagli C , Archetti M, Mignot I et al. Combining antibiotics with antivirulence compounds can have synergistic effects and reverse selection for antibiotic resistance in *Pseudomonas aeruginosa*. PLoS Biol 2020;18:e3000805. 32810152 10.1371/journal.pbio.3000805 PMC7433856

[85] Scholthof KBG . The disease triangle: pathogens, the environment and society. Nat Rev Microbiol 2007;5:152–6. 17191075 10.1038/nrmicro1596

[86] Yu K , Pieterse CMJ, Bakker PAHM et al. Beneficial microbes going underground of root immunity. Plant Cell Environ 2019;42:2860–70. 31353481 10.1111/pce.13632 PMC6851990

[87] Hacquard S , Spaepen S, Garrido-Oter R et al. Interplay between innate immunity and the plant microbiota. Annu Rev Phytopathol 2017;55:565–89. 28645232 10.1146/annurev-phyto-080516-035623

[88] Farace G , Fernandez O, Jacquens L et al. Cyclic lipopeptides from *Bacillus subtilis* activate distinct patterns of defence responses in grapevine. Mol Plant Pathol 2014;16:177–87. 25040001 10.1111/mpp.12170 PMC6638491

[89] Deravel J , Lemière S, Coutte F et al. Mycosubtilin and surfactin are efficient, low ecotoxicity molecules for the biocontrol of lettuce downy mildew. Appl Microbiol Biotechnol 2014;98:6255–64. 24723290 10.1007/s00253-014-5663-1

[90] Leclère V , Béchet M, Adam A et al. Mycosubtilin overproduction by *Bacillus subtilis* BBG100 enhances the organism's antagonistic and biocontrol activities. Appl Environ Microbiol 2005;71:4577–84. 16085851 10.1128/AEM.71.8.4577-4584.2005 PMC1183317

