# Supplementary material for: Depletion of protective microbiota promotes the incidence of fruit disease
Source: ISME J. 2024 May 1;18(1):wrae071. doi: 10.1093/ismejo/wrae071 (PMC11654636; doi:10.1093/ismejo/wrae071)
Supplement: Supplementary_Information_wrae071 [file supplementary_information_wrae071.docx]

**Supplementary Information**

**Depletion of the protective microbiota promotes the incidence of fruit disease**

Xue Luo^1,2^, Kai Sun^1,2^，Hao-Ran Li^1,2^, Xiang-Yu Zhang^1^, Yi-Tong Pan^1^, De-Lin Luo^1^, Yi-Bo Wu^1^, Hui-Jun Jiang^1^, Xiao-Han Wu^1^, Chen-Yu Ma^1^, Chuan-Chao Dai^1^, Wei Zhang^1^*****

^1^Jiangsu Key Laboratory for Microbes and Functional Genomics, Jiangsu Engineering and Technology Research Center for Industrialization of Microbial Resources, College of Life Sciences, Nanjing Normal University, Jiangsu Province, China

^2^These authors contributed equally: Xue Luo, Kai Sun, and Hao-Ran Li

*Corresponding to: zhwnjnu@163.com

**Supplementary Methods**

**Soil physiochemical properties analysis**

The soil pH, total nitrogen (TN), total phosphorus (TP), total potassium (TK), available nitrogen (AP), available phosphorus (AP), available potassium (AK), and organic matter (OM) were measured according to standard methods [1].

***In vitro* and *in vivo* analysis of fungal isolates on pod disease**

To test whether the isolated fungal strains cause pod disease, both *in vitro* and *in vivo* assays were performed. For the *in vitro* assay, healthy pods were surface-sterilized by immersion in 70% (v/v) ethanol for 1min, 1.5% (v/v) sodium hypochlorite solution for 15 min, followed by washed three times with sterile water. To check if the pods were well surface-sterilized, the pods and 100 µl of the remaining washing water were placed on TSA and PDA plates. Pods having no colony growth will be used for downstream treatment. Each pod was inoculated with 1 ml of spore suspension (1 × 10^7^ conidia ml^-1^). Spores of fungal isolates were collected from two-week-old fungal cultures, quantified by hemocytometer, then diluted to 10^7^ conidia ml^-1^. Control received same amount of sterile water. Each treatment included four plates and each plate contained five pods. After incubation at 28°C for 7 days, the pods were collected for disease inspection.

For the *in vivo* assay of fungal isolates on pod disease, peanut seedlings were grown in pots (28 cm diameter, 22 cm height) with the sterile commercial substrate in a glasshouse with day: 25-30°C; night: 20-25°C, 60 ± 5% relative humidity and natural sunlight. When the peanut pegs were formed and began to penetrate into soil (60 days after sowing), two Erlenmeyer flasks (50 ml) with sterile commercial substrate were inserted into pot to allow peg grow in. Each Erlenmeyer flask contained one peg and each pot included two Erlenmeyer flasks. Each Erlenmeyer flask received fungal spore suspension (10^6^ conidia per g of substrate). Control received same amount of sterile water. Spores of fungal isolates were collected from two-week-old fungal cultures, quantified by hemocytometer, then diluted to 10^6^ conidia per g of sterile substrate. The sterile water without fungal conidia was used as the negative control. Five individual replicates were established for each treatment. At 30 days after inoculation, the pods were collected for disease inspection.

**Assays of various plant-beneficial traits**

**Measurements of bacterial siderophores** Briefly, 5 µl of fresh BcGE1, BaGE2, BaGE3, and SynCom1 culture were spotted on the center of plates containing Chrome azurol sulfonate (CAS) agar medium [2]. The plates were inoculated at 30°C for 3 days. Production of siderophores was visualized by an orange color in the form of a hole surrounding the bacterial colony. The siderophores production efficiency is measured as Solubilizing efficiency (SE) = (Z-C)/C, where Z = solubilization zone (mm), C = colony diameter (mm).

**Phosphate solubilization assay.** Briefly, 5 µl of fresh of BcGE1, BaGE2, BaGE3, and SynCom1 culture were spotted on the center of plates containing Pikovskaya’s agar medium (0.5 g/L yeast extract, 10 g/L dextrose, 5.0 g/L Ca_3_(PO4)_2_, 0.5 g/L (NH_4_)_2_SO_4_, 0.2g/L KCl, 0.1g/L MgSO_4_.7H_2_O, 0.0001 g/L MnSO_4_, 0.0001 g/L FeSO_4_.7H_2_O, and 20 g/L agar). The plates were inoculated at 30°C for 3 days. Phosphate solubilization was visualized by a halo zone surrounding the bacterial colony. The phosphate solubilizing efficiency is measured as Solubilizing efficiency (SE) = (Z-C)/C, where Z = solubilization zone (mm), C = colony diameter (mm).

**Potassium solubilization assay** Briefly, 5 µl of fresh of BcGE1, BaGE2, BaGE3, and SynCom1 culture were spotted on the center of plates potassium-releasing bacteria medium (1.0 g/L potassium-feldspar powder, 5.0 g/L sucrose, 0.2 g/L MgSO_4_.7H_2_O, 2.0 g/L Na_2_HPO_4_, 0.1 g/L CaCO3, 0.05 g/L FeCl_3_). The plates were inoculated at 30°C for 5 days. Potassium solubilization was visualized by a halo zone surrounding the bacterial colony. The potassium solubilizing efficiency is measured as Solubilizing efficiency (SE) = (Z-C)/C, where Z = solubilization zone (mm), C = colony diameter (mm).

**Measurements of bacterial-produced auxin** The BcGE1, BaGE2, BaGE3, and SynCom1 were cultured in liquid medium (DF minimal salt) supplemented with 5 mM L-tryptophan. The DF minimal salt medium contained following components, 4.0 g/L KH_2_PO_4_, Na_2_HPO_4_, 0.2 g/L MgSO_4_.7H_2_O, 1 mg/L FeSO_4_.7H_2_O, 10 µg/L H_3_BO_3_, 10 µg/L MnSO_4_, 70 µg/L ZnSO_4_, 50 µg/L CuSO_4_, 10 µg MoO_3_, 2 g/L glucose, 2 g/L gluconic acid, and 2 g/L citric acid. After 3 days of incubation at 30°C with shaking at 180 rpm, the bacterial culture was centrifuged at 5000 × g for 10 min, the supernatant was mixed (1:1, v/v) with Salkowski reagent (12 g of FeCl_3_ per 1 L in 7.9 M H_2_SO_4_), and incubated in dark at room temperature for 30 min. The absorbance was measured at 520 nm with a SpectraMax M2 Microplate reader (Molecular Devices, USA). The concentration of indole-3-acetic acid (IAA) produced by the bacteria was determined from a standard curve generate using a standard solution of commercial IAA.

**Measurements of bacterial ACC deaminase activities** The bacterial 1-aminocyclopropane-1-carboxylate (ACC) deaminase activities were determined as previously reported [3]. Briefly, 100 µl of BcGE1, BaGE2, BaGE3, and SynCom1 were spread on DF minimal salt agar medium. The medium also contained 3 mM filter-sterilized ACC as the sole source of nitrogen. The plates were inoculated at 30°C for 3 days. The strains were able to grow on the medium containing ACC were recorded as strains with ACC deaminase activity. The efficiency of ACC deaminase activity was expressed as relative bacteria growth ratio by comparing the finial bacterial CFUs to initial bacterial CFUs.

**Bacterial-fungal interaction analysis**

Spores of *A. flavus* GE1 and *A. niger* GE2 were collected from three-week-old fungal cultures, and re-suspended in 10 ml sterile water, and centrifuged for 5 min at 2000 rpm. After three washes with sterile water, spore concentration was adjusted to 1 × 10^6^ spores/ml in liquid tryptic soy broth (TSB) medium (20%). Bacterial cells of *B. cereus* GE1, *B. amyloliquefaciens* GE2, *B. altitudinis* GE3, *B. halotolerans* GE7, *Paenibacillus* sp. GE8, and *B. siamensis* GE10 were collected from overnight growth bacterial cultures, and resuspended in TSB (20%), and centrifuged for 10 min at 4000 g. After three washes with TSB (20%), bacterial concentration was adjusted to OD600 = 0.1 in TSB (20%). To conduct the SynCom treatment, equal volume of corresponding strain suspension cultures were mixed [4], and then the OD_600_ of SynCom was adjusted to 0.1.

Two 96-well optical bottom plates (Corning, NY, USA) were used for the bacterial-fungal interaction analysis. Plate A contained fungal spores with or without a single strain or SynCom. Plate B contained a single strain or SynCom to assess bacterial autofluoresence intensity. Plate A was filled with 150 μl of TSB (20%) or 160 μl of TSB (20%) for the control without bacteria. Plate B was filled with 190 μl of TSB (20%). Then, 40 μl of fungal spores were inoculated into the all wells of plate A and 10 μl of single strain or SynCom was inoculated to both plates, except for the control in plate A. After 48 hours of incubation at 25°C, plates were washed away with a multichannel pipet and the wells were further washed two times with 200 μl of phosphate buffer saline (PBS, pH 7.2). All wells in both plates were incubated with 100 μl of PBS supplemented with 1 μg/mL of Wheat Germ Agglutinin (WGA) Alexa fluor 488 conjugated (Invitrogen/Molecular Probes, Eugene, USA) overnight at 4°C. The solution was washed away and the wells were washed two times with 200 μl of PBS (pH 7.2). The fluorescence intensity was measured using a SpectraMax M2 Microplate reader (Excitation/Emission 490/530, Molecular Devices, USA). Log2-transformed relative fluorescence values, reflecting the ability of single *Bacillus* strain and SynCom to restrict fungal growth [5,6].

**Effects of *Bacillus* isolates against *Aspergillus* on detached fruits or tubers**

Healthy maize kernels (Xianyu 335), potato tubers (Favorita 15), apple fruits (*Malus domestica* cv. Gala), and strawberry fruits (Fragaria × ananassa) were purchased from local market. For maize kernels and strawberry fruits, each maize kernel and strawberry fruit was coinoculated with 0.25 ml of spore suspension (1 × 10^7^ conidia/ml) and 0.25 ml of sterile water, *Bacillus* isolates, SynCom1 or SynCom2. For potato tubers and apple fruits, a hole (Ø 0.7 cm) was firstly made on the potato tubers and apple fruit. Next, 0.25 ml of spore suspension (1 × 10^7^ conidia/ml) and 0.25 ml of sterile water, *Bacillus* isolates, SynCom1 or SynCom2 were co-inoculated into the hole. Spores of fungal isolates were collected from two-week-old fungal cultures, quantified by hemocytometer, then diluted to 10^7^ conidia/ml. Each treatment was performed in 4 individual replicates. After incubation at 28°C for 5 days, the fruits and tubers were collected for *Aspergillus* biomass quantification. Specific primer pairs of *A. flavus* GE1 (Fla-F/Fla-R) [7] and *A. niger* GE2 (An-F/An-R) [8] were used to quantify the *Aspergillus* amount on the surface of maize kernels, potato tubers, apple fruits, and strawberry fruits with quantitative PCR with a 7500 Real-Time PCR System (Applied Biosystems, Pleasanton, CA, USA). Gene copy numbers were expressed as log_10_ values. The primers are listed in Table S2.

**ROS visualization**

For visualization of ROS, the NBT stain was prepared from 2.5 mM NBT chloride and 5 mM (*N*-morpholino) propane sulfonate-NaOH buffer at pH 7.6 [9]. The NBT reacts with O_2_^.-^ to form a blue precipitate. The solution was shielded from light to prevent photo-oxidation. Plates were flooded with 15 ml of NBT stain, and then incubated for 20 min in the dark, decanted to remove excess stain, and imaged using a stereomicroscope. The hyphae proximate and distal to the bacterial stream were observed using a light microscope.

**Reference**

1. Zhang W, Bai YN, Sun K, Zhang W, Dai CC. Reduced pollen activity in peanut (*Arachis hypogaea* L.) by long-term monocropping is linked to flower water deficit. Plant Soil. 2023;482:427-450
2. Alexander DB & Zuberer DA. Use of chrome azurol S reagents to evaluate siderophore production by rhizosphere bacteria. Biol Fertil Soils. 1991;12:39-45
3. He D, Singh SK, Peng L, Kaushal R, Vílchez JI, Shao C, et al. Flavonoid-attracted *Aeromonas* sp. from the Arabidopsis root microbiome enhances plant dehydration resistance. ISME J. 2022;16:2622-2632
4. Zhou X, Wang J, Liu F, Liang J, Zhao P, Tsui CKM, et al. Cross-kingdom synthetic microbiota supports tomato suppression of Fusarium wilt disease. Nat Commun. 2022;13:7890
5. [Figueroa-López](https://onlinelibrary.wiley.com/authored-by/Figueroa%E2%80%90L%C3%B3pez/Alejandro+Miguel) AM, Cordero-Ramírez JD, Quiroz-Figueroa FR, Maldonado-Mendoza IE. A high-throughput screening assay to identify bacterial antagonists against *Fusarium verticillioides*. J Basic Microbiol. 2014;54 (Suppl 1):S125-S133
6. Durán, P. Thiergart T, Garrido-Oter R, Agler M, Kemen E, Schulze-Lefert P, et al. Microbial interkingdom interactions in roots promote Arabidopsis survival. Cell. 2018;175:973-983
7. Al-Shuhaib MBS, Albakri AH, Alwan SH, Almandil NB, AbdulAzeez S, Francis Borgio J. Optimal pcr primers for rapid and accurate detection of *Aspergillus flavus* isolates. Microb. Pathog. 2018;116:351-355.
8. Peterson SW. Phylogenetic analysis of *Aspergillus* species using DNA sequences from four loci. Mycologia, 2008;100:205-226.
9. Yu GH, Chi ZL, Kappler A, Sun FS, Liu CQ, Teng H, et al. Fungal nanophase particle catalyze iron transformation for oxidative stress removal and iron acquisition. Curr Biol. 2020;30:2943-2950

**Supplementary figures**

**
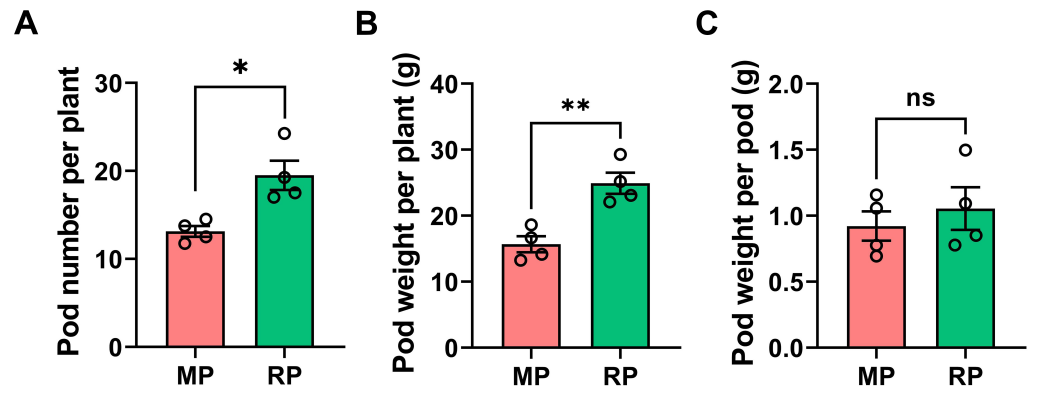
Supplementary Figure 1 Monocropping reduces pod number and pod weight per peanut plant. A** MP reduces pod number per peanut. **B** MP reduces pod weight per peanut. **C** MP did not significantly influence single pod weight. Data are the mean ± SEM (n = 4 plots per treatment). The asterisk indicates a significant difference between monocropping and rotation treatments according to Student’s *t* test (**P* < 0.05, ***P* < 0.01). ns indicates a nonsignificant difference. MP, monocropping; RP, rotation.

**
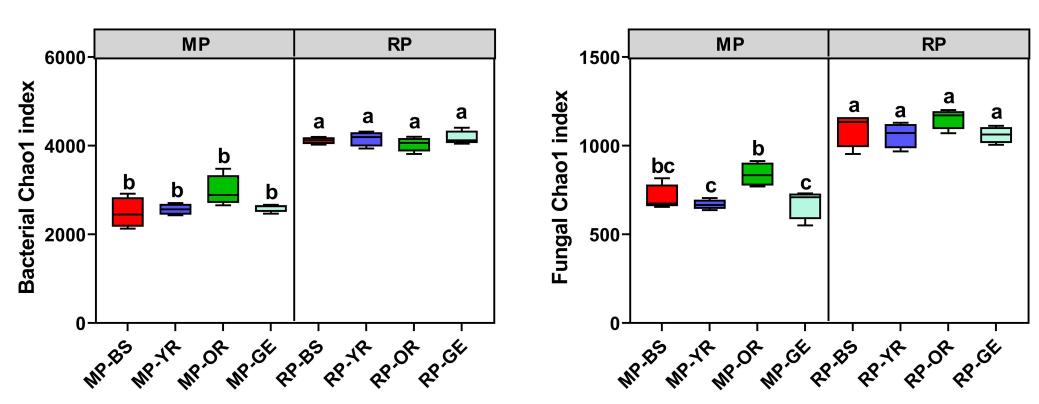
**

**Supplementary Figure 2 The bacterial and fungal Chao1 index analysis of samples from pot experiment 1.** Bacterial and fungal Chao1 index analysis of MP-BS, MP-YR, MP-OR, MP-GE, RP-BS, RP-YR, RP-OR, and RP-GE samples. Boxplots indicate median (middle line), 25th, 75th percentiles (box), and maximum and minimum values (whiskers) (n = 4 individual replicates). Different letters indicate significant differences (**P* < 0.05, one-way analysis of variance followed by Tukey’s honest significant difference test). MP-BS, monocropped-bulk soil; MP-YR, monocropped-young rhizosphere soil; MP-OR, monocropped-old rhizosphere soil; MP-GE, monocropped-geocarposphere soil; RP-BS, rotated-bulk soil; RP-YR, rotated-young rhizosphere soil; RP-OR, rotated-old rhizosphere soil; RP-GE, rotated-geocarposphere soil.

**
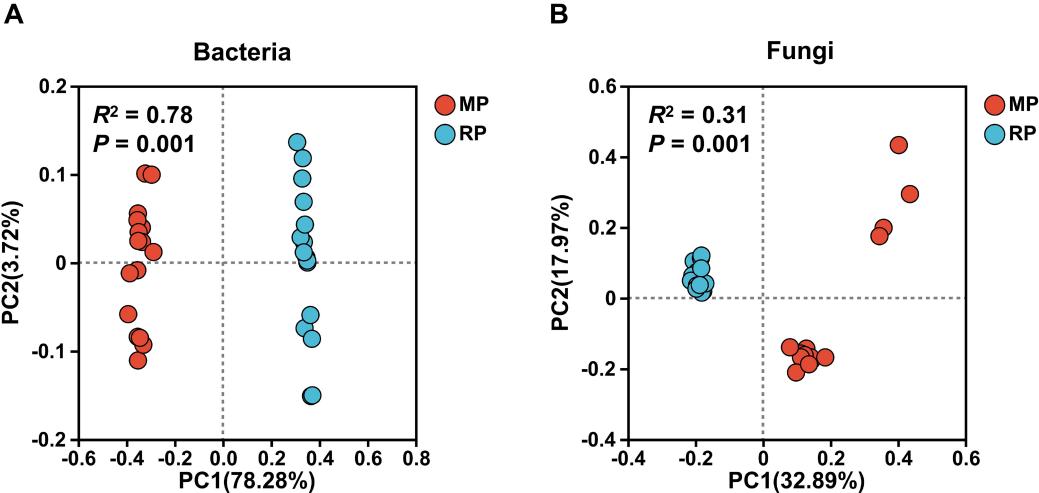
**

**Supplementary Figure 3 PCoA (based on the relative abundance of OTUs) analysis of Bray-Curtis distances of MP and RP samples.** **A** PCoA (based on the relative abundance of bacterial OTUs) of Bray-Curtis distances of MP and RP samples. **B** PCoA (based on the relative abundance of fungal OTUs) of Bray-Curtis distances of MP and RP samples. PERMANOVA was performed using the adonis function from the R package.

**
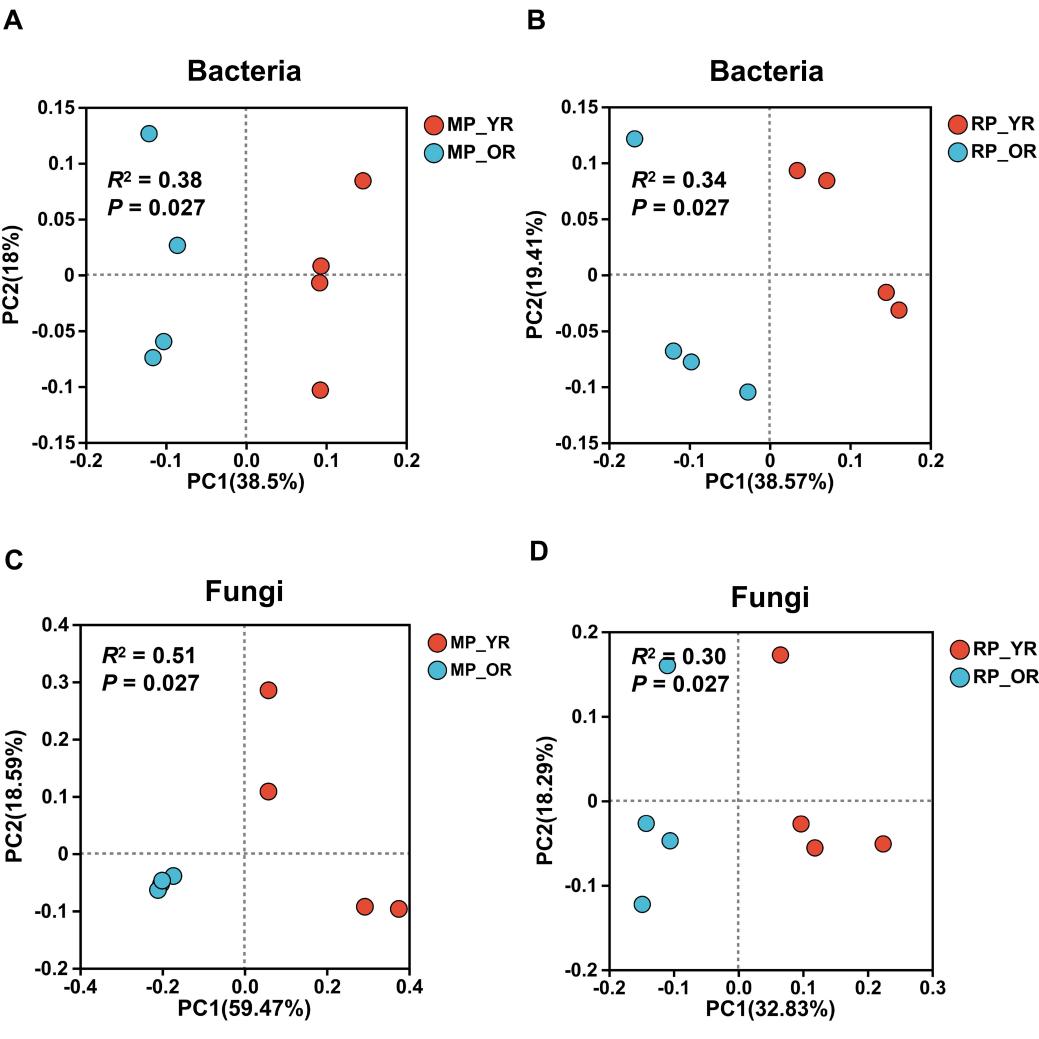
**

**Supplementary Figure 4 PCoA (based on the relative abundance of OTUs) analysis of Bray-Curtis distances of YR and OR samples under MP and RP conditions.** **A, B** PCoA (based on the relative abundance of bacterial OTUs) of Bray-Curtis distances of YR and OR samples under MP **(A)** and RP **(B)** conditions. **C, D** PCoA (based on the relative abundance of fungal OTUs) of Bray-Curtis distances of YR and OR samples under MP **(C)** and RP **(D)** conditions. PERMANOVA was performed using the adonis function from the R package.

**
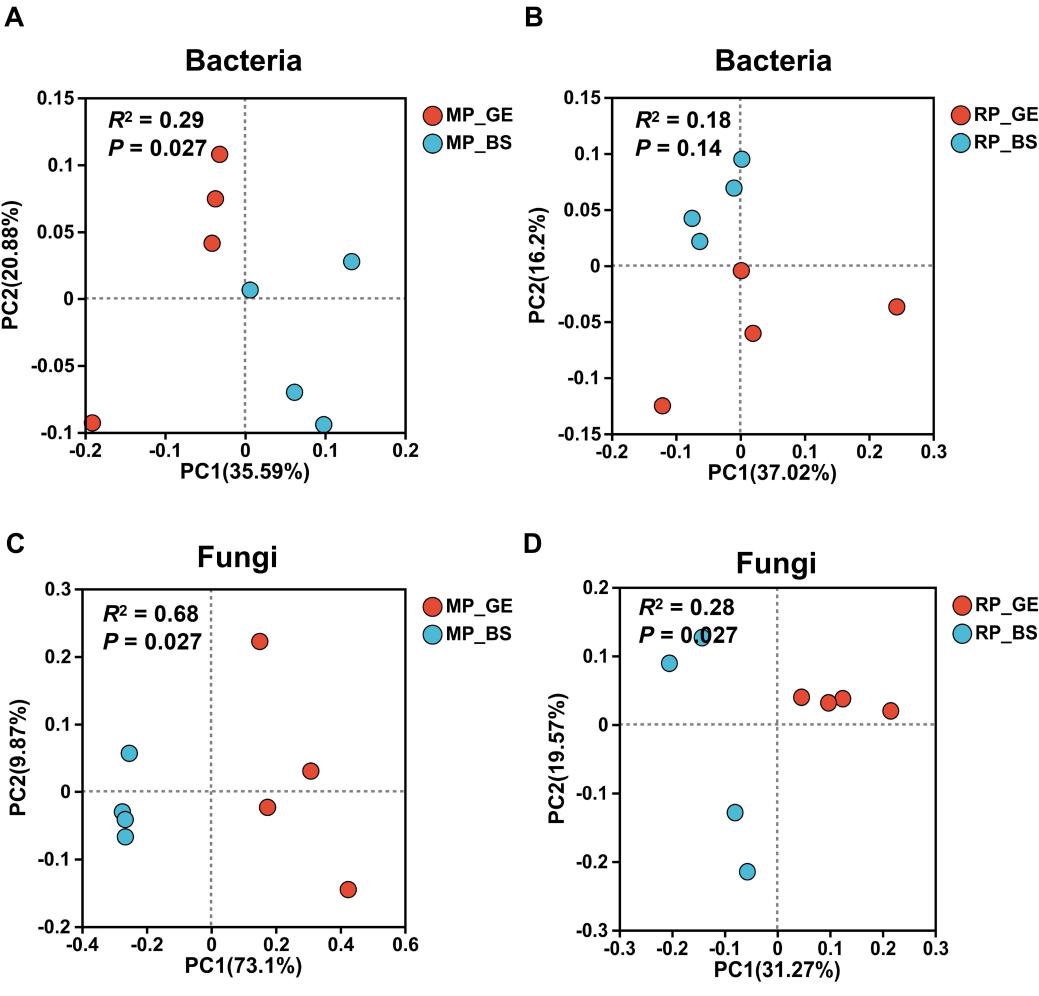
**

**Supplementary Figure 5 PCoA (based on the relative abundance of OTUs) analysis of Bray-Curtis distances of GE and BS samples under MP and RP conditions.** **A, B** PCoA (based on the relative abundance of bacterial OTUs) of Bray-Curtis distances of GE and BS samples under MP **(A)** and RP **(B)** conditions. **C, D** PCoA (based on the relative abundance of fungal OTUs) of Bray-Curtis distances of GE and BS samples under MP **(C)** and RP **(D)** conditions. PERMANOVA was performed using the adonis function from the R package.


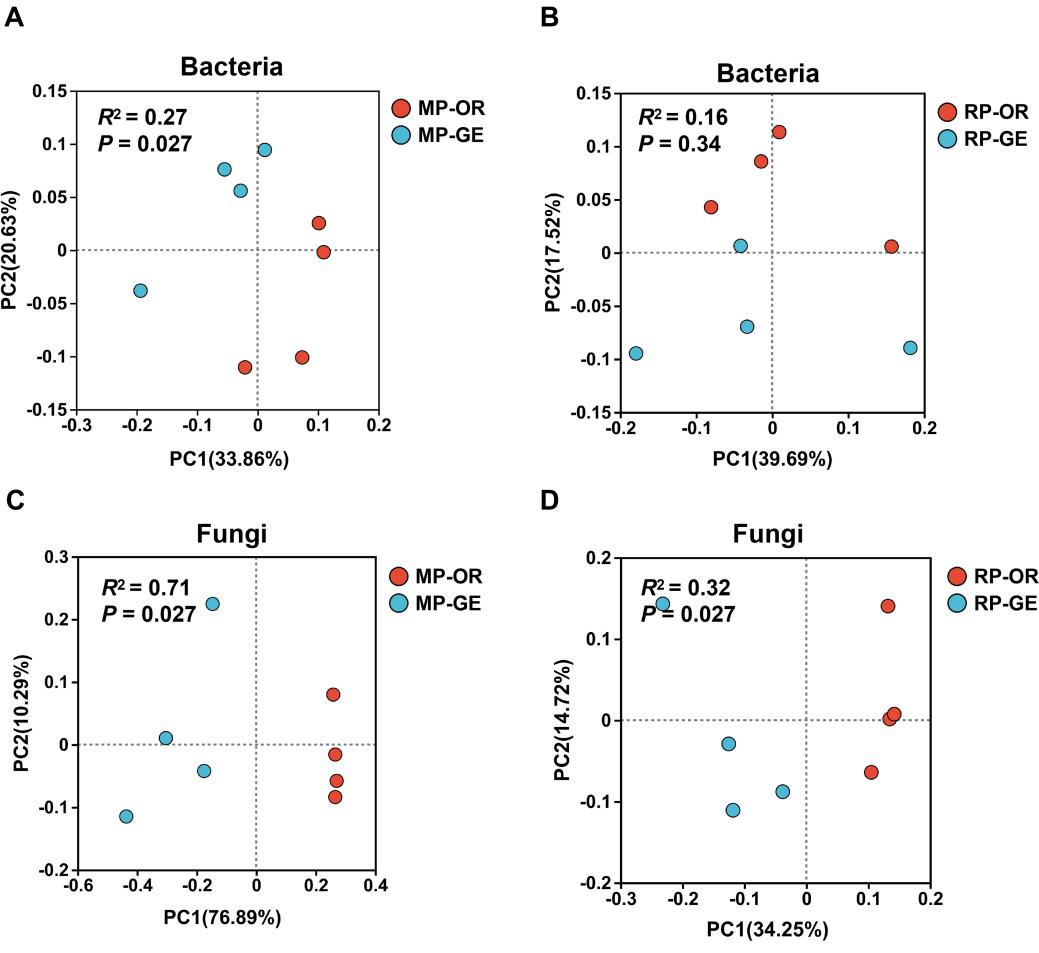


**Supplementary Figure 6 PCoA (based on the relative abundance of OTUs) analysis of Bray-Curtis distances of OR and GE samples under MP and RP conditions.** **A, B** PCoA (based on the relative abundance of bacterial OTUs) of Bray-Curtis distances of OR and GE samples under MP **(A)** and RP **(B)** conditions. **C, D** PCoA (based on the relative abundance of fungal OTUs) of Bray-Curtis distances of OR and GE samples under MP **(C)** and RP **(D)** conditions. PERMANOVA was performed using the adonis function from the R package.


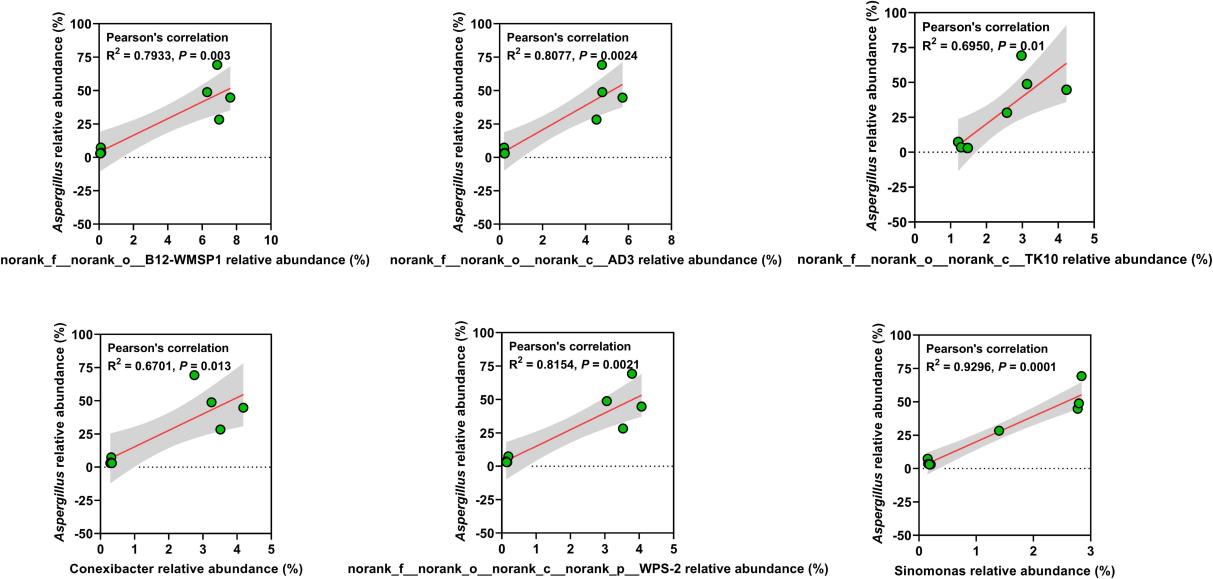


**Supplementary Figure 7 Pairwise correlation analyses between the relative abundance of six bacterial genera and the relative abundance of *Aspergillus*.**


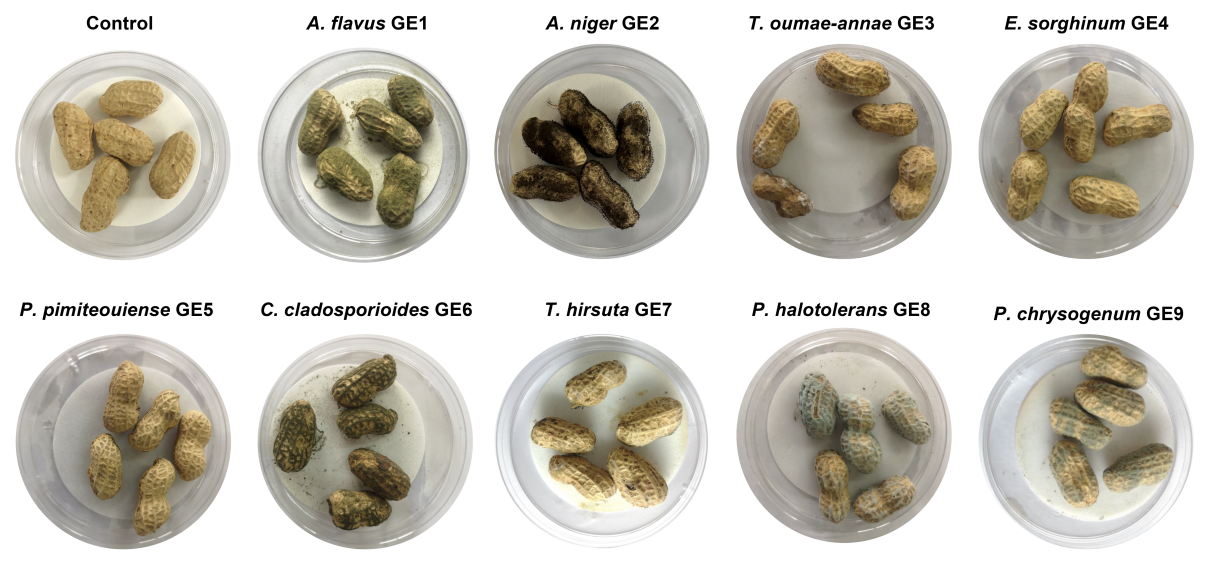
**Supplementary Figure 8 *In vitro* analysis of fungal isolates on pod disease.** Each pod was inoculated with 1 ml of spore suspension (1 × 10^7^ conidia/ml). Control received same amount of sterile water. The pods were then placed on plates, and each plate contained 5 pods. Each treatment was performed in 4 individual replicates. After incubation at 28°C for 7 days, the pods were collected for disease inspection. *A. flavus* GE1, *A. niger* GE2, *T. oumae-annae* GE3, *C. cladosporiodies* GE6, *P. halotelerans* GE8, and *P. chrysogenum* GE9 clearly caused pod diseases.


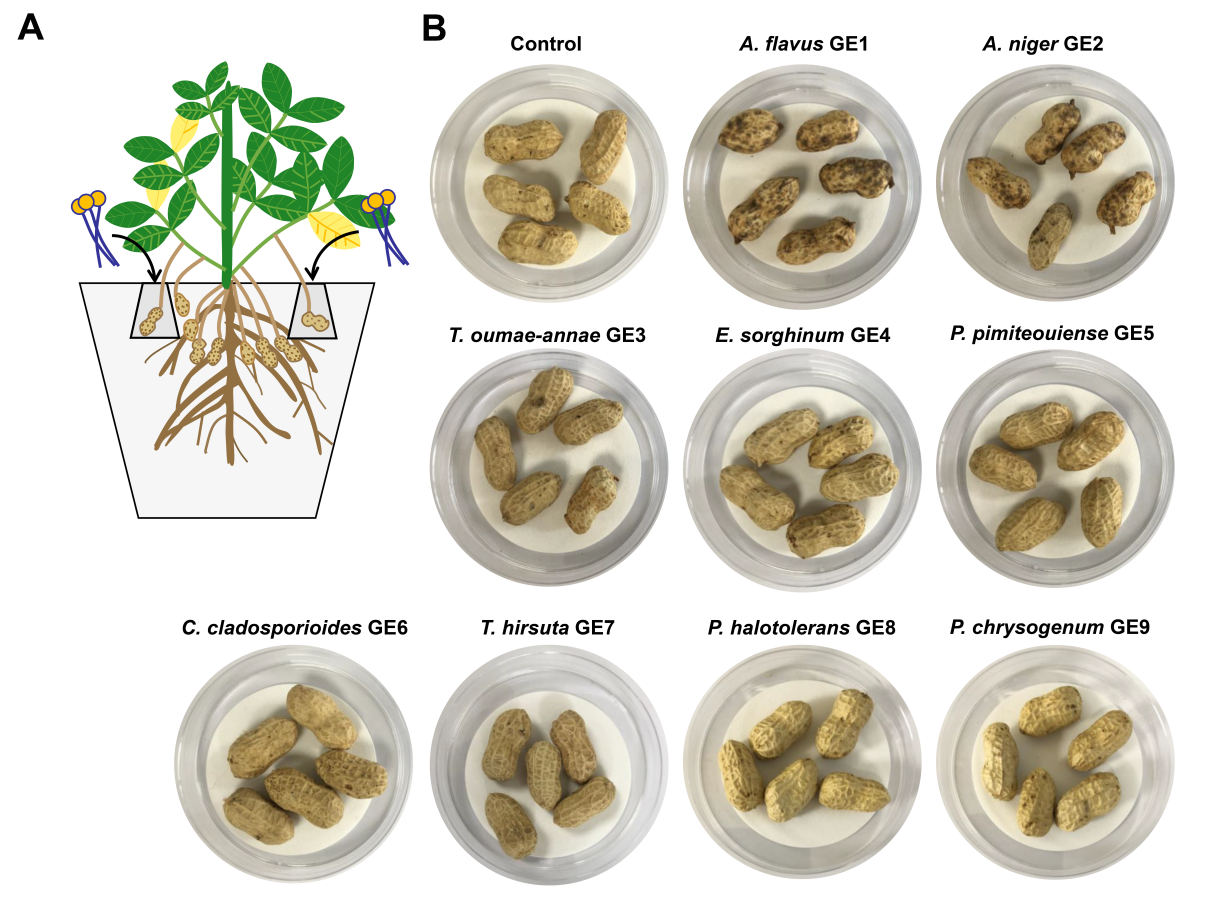
**Supplementary Figure 9 *In vivo* analysis of fungal isolates on pod disease. A** Peanut seedling was grown in pot (28 cm diameter, 23 cm height) with the sterile commercial substrates in a glasshouse with 30/24°C temperature regime and nature sunlight. When the peanut pegs were formed and began to penetrate into soil (60 days after sowing), two Erlenmeyer flask (50 ml) with sterile commercial substrates were inserted into pots to allow peg grow in. Each Erlenmeyer flask contained one peg. Each Erlenmeyer flask received 2 ml of spore suspension (1 × 10^7^ conidia ml^-1^). Control received same amount of sterile water. Five individual replicates were established for each fungal strain. At 30 days after inoculation, the pods were collected for disease inspection. **B** *A. flavus* GE1 and *A. niger* GE2 clearly caused pod disease.

**
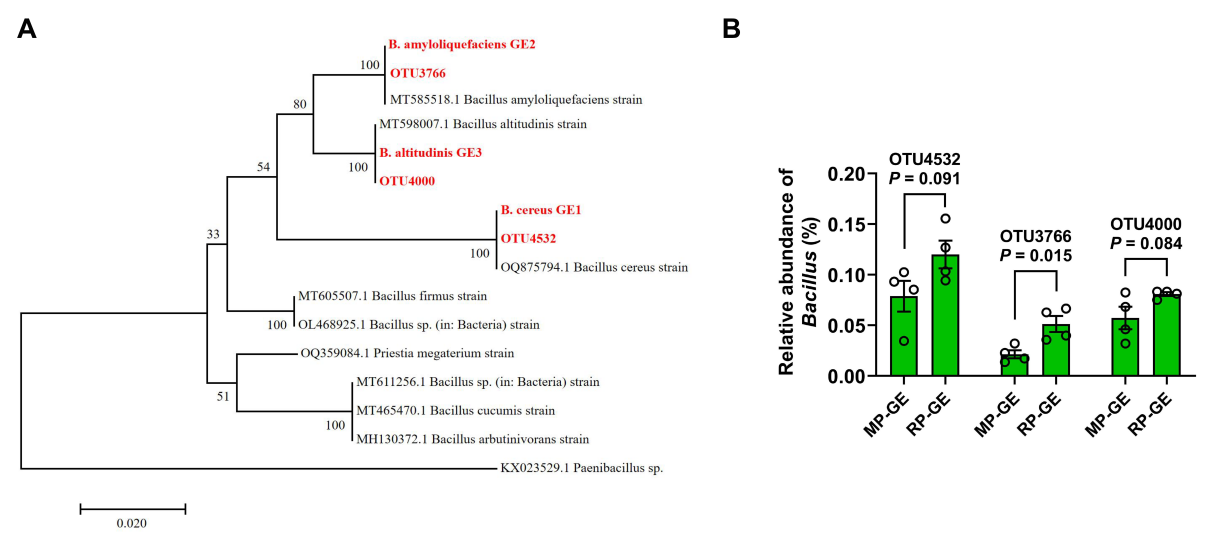
Supplementary Figure 10 (A)** Phylogenetic reconstructions of BcGE1, OTU4532, BaGE2, OTU3766, BaGE3, and OTU4000. Each independent phylogenetic reconstruction included best match sequences obtained from NCBI database for taxonomical inferences. Numbers on each node are bootstrap values of 1000 replicates. **(B)** Relative abundance of OTU4532, OTU3766, and OTU4000 in MP-GE and RP-GE samples. Data are the mean ± SEM (n = 4 individual replicates). Significant differences were calculated with one-way analysis of variance followed by Tukey’s honest significant difference test.

**
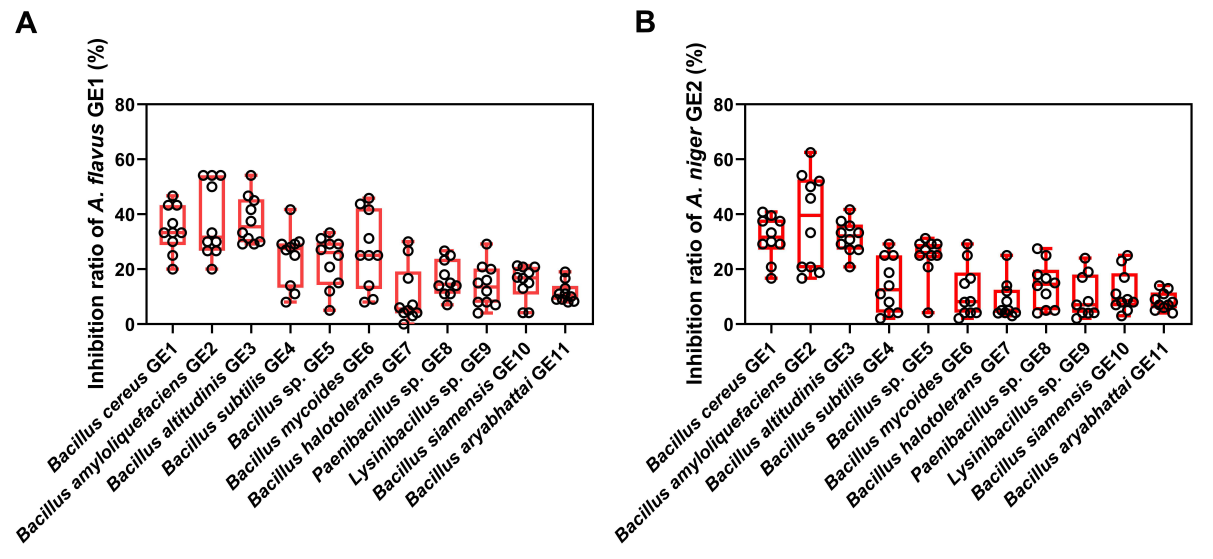
Supplementary Figure 11 The inhibition ratio of *Bacillus* isolates on** ***A. flavus* GE1 and *A. niger* GE2. A** The inhibition ratio of *Bacillus* isolates on *A. flavus* GE1. **B** The inhibition ratio of *Bacillus* isolates on *A. niger* GE2. The boxplots show the data from 10 independent replicates. Boxplots indicate median (middle line), 25th, 75th percentiles (box), and maximum and minimum values (whiskers) as well as outliers (single points).

**Supplementary Figure
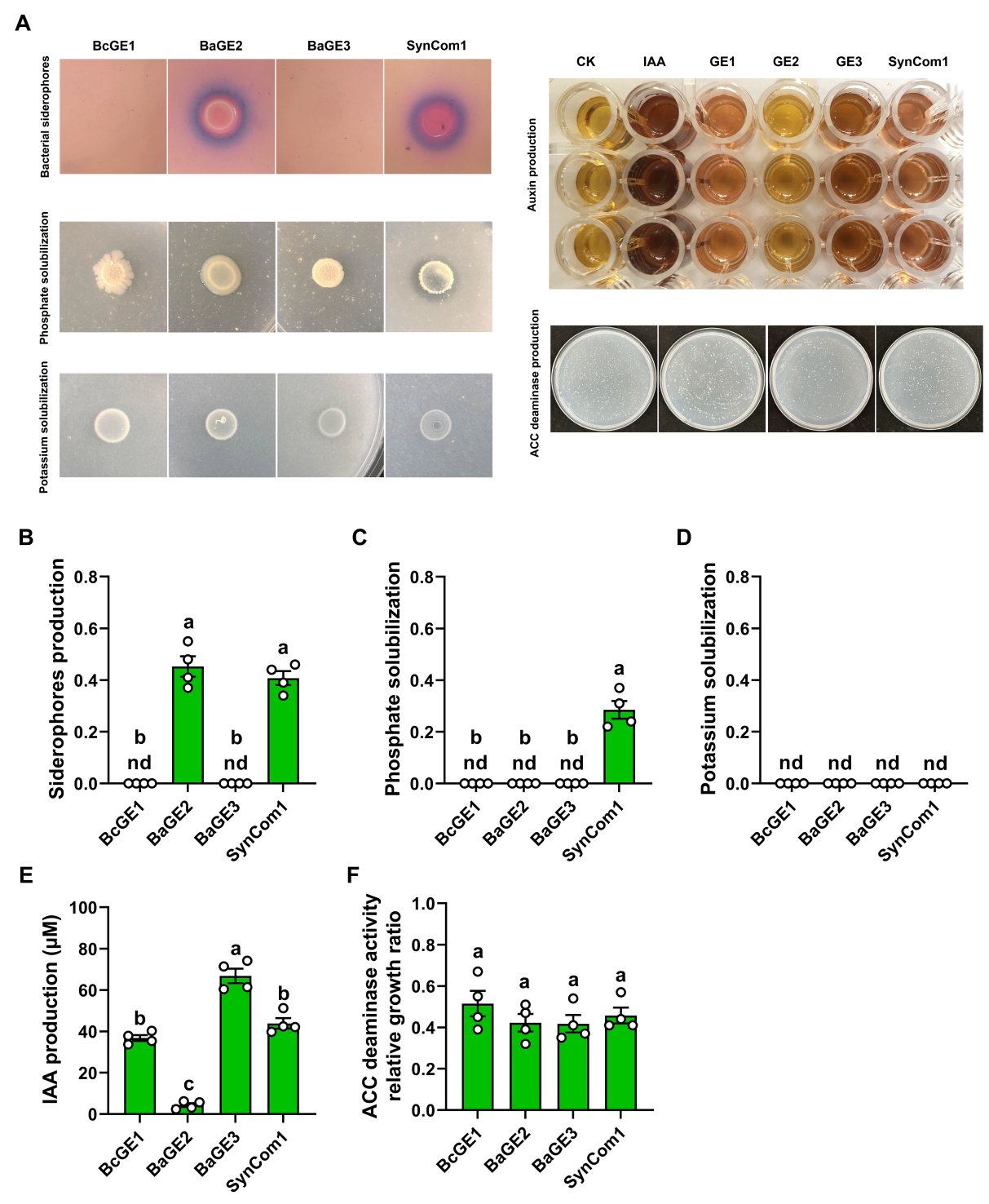
12 Plant beneficial traits of BcGE1, BaGE2, BaGE3, and SynCom1. A** Representative images showing plant beneficial traits, including siderophore production, phosphate solubilization, potassium solubilization, auxin secretion and ACC deaminase production of BcGE1, BaGE2, BaGE3, and SynCom1. **B-F** The siderophore production **(B)**, phosphate solubilization **(C)**, potassium solubilization **(D)**, auxin production **(E)**, and ACC deaminase production **(F)** of BcGE1, BaGE2, BaGE3, and SynCom1. IAA, indole-3-acetic acid; ACC, 1-aminocyclopropane-1-carboxylate. Data are the mean ± SEM (n = 4 individual replicates). Different letters indicate significant differences (**P* < 0.05, one-way analysis of variance followed by Tukey’s honest significant difference test).

**
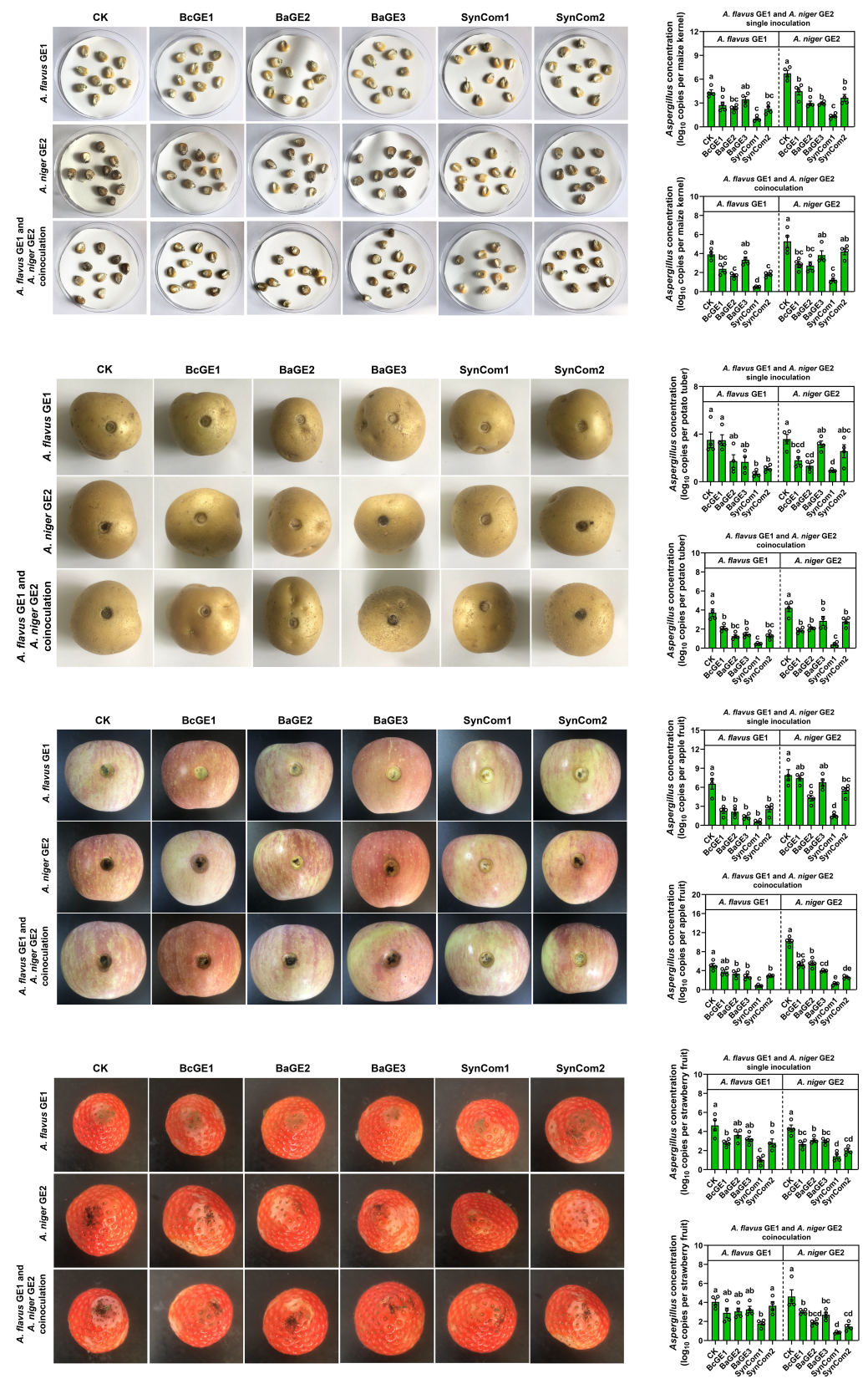
Supplementary Figure 13 Effects of *Bacillus* isolates against *Aspergillus* on detached fruits or tubers.** Quantification of *A. flavus* GE1 and *A. niger* GE2 biomass on the surface of maize kernels, potato tubers, apple fruits, and strawberry fruits with sterile water, BcGE1, BaGE2, BaGE3, SynCom1, and SynCom2 coinoculation. Data are the mean ± SEM (n = 4 individual replicates). Different letters indicate significant differences among treatments (**P* < 0.05, one-way analysis of variance followed by Tukey’s honest significant difference test).


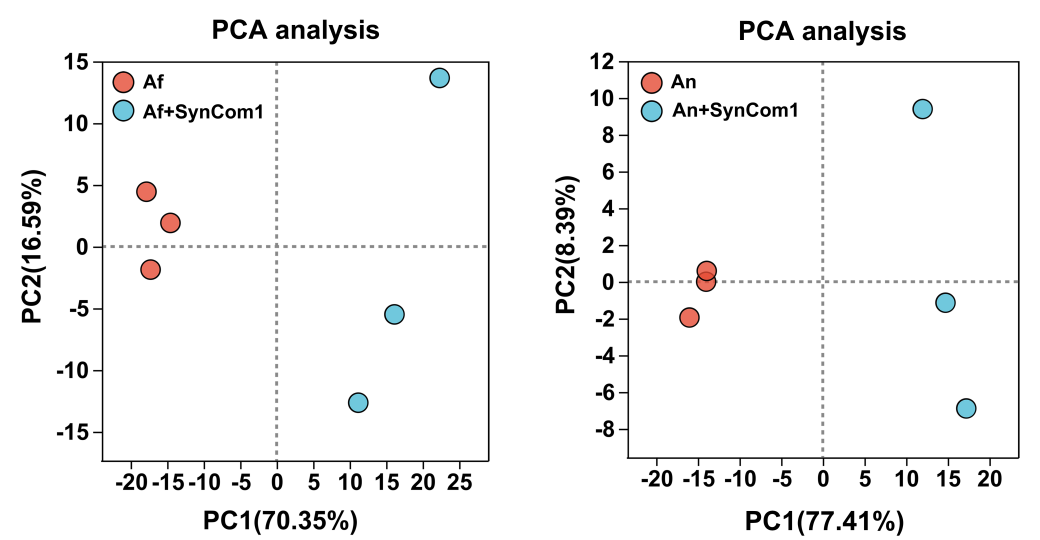
**Supplementary Figure 14 PCA analysis showing the divergence of the respective transcriptome from different treatments.** Af, *Aspergillus flavus*; An, *Aspergillus niger*.

**
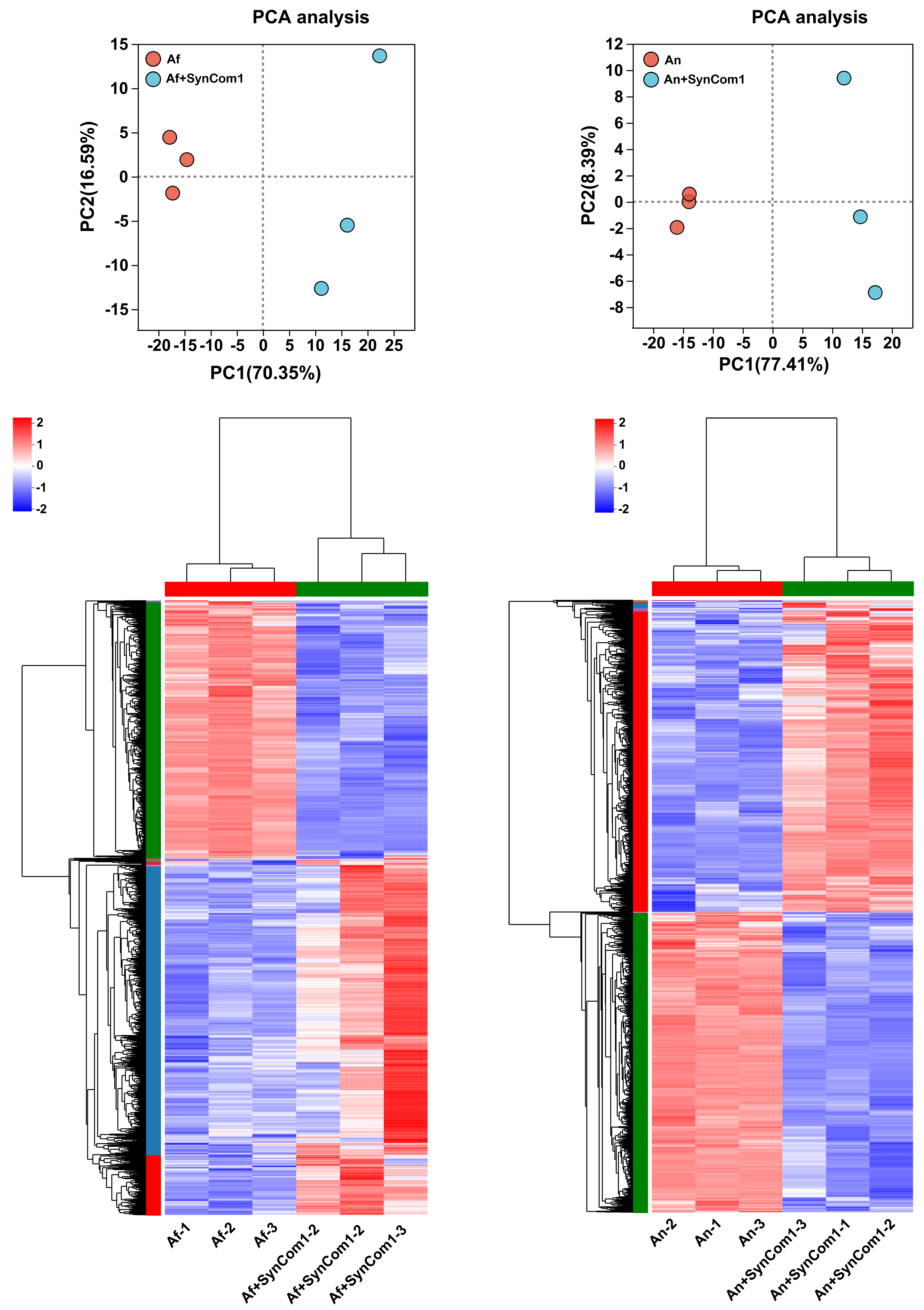
Supplementary Figure 15 Heatmap showing the gene expression patterns of *Aspergillus flavus* and *Aspergillus niger* from different treatments.** Top, sample tree; left; gene tree; bottom, treatments; Af, *Aspergillus flavus*; An, *Aspergillus niger*.

**
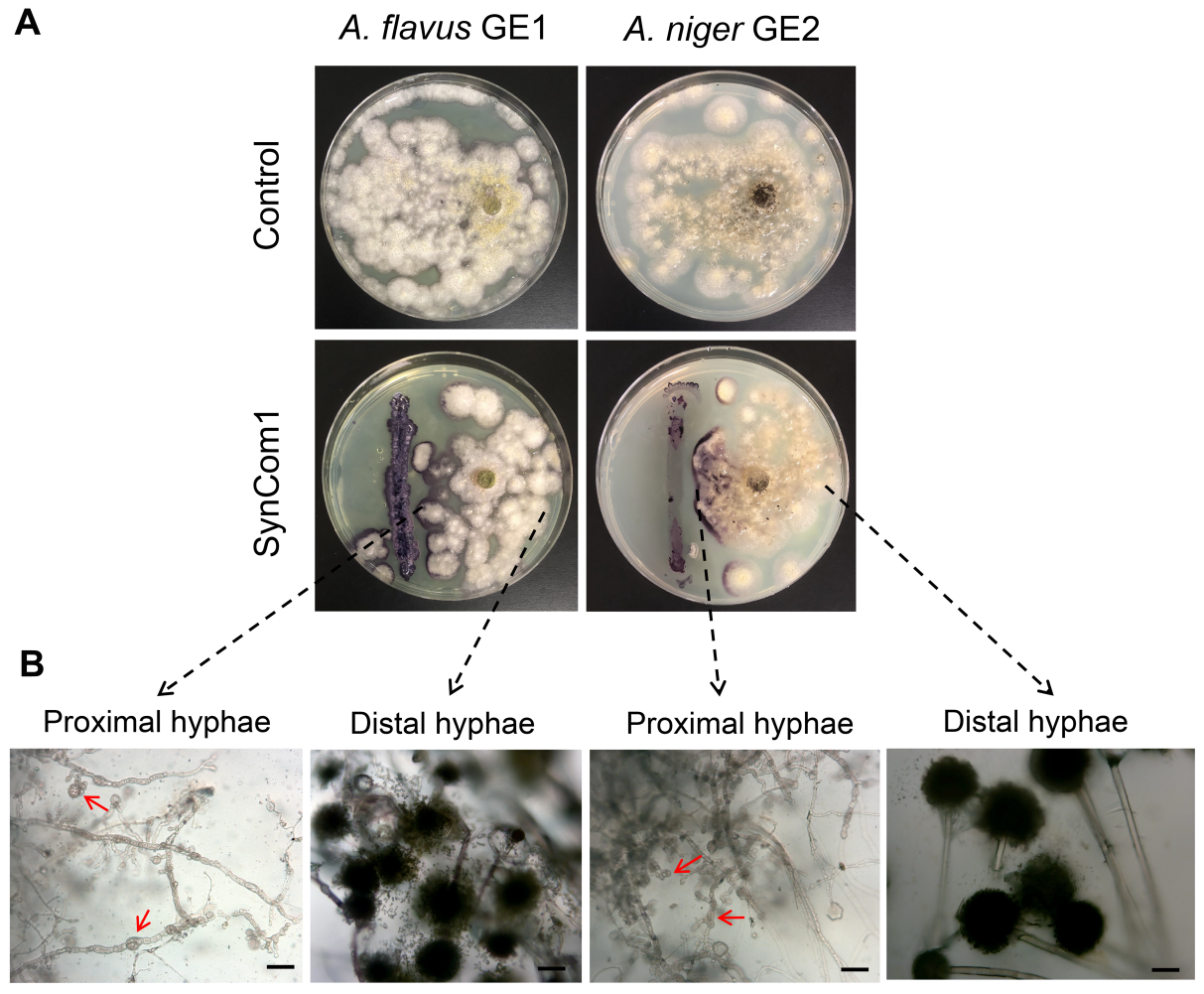
Supplementary Figure 16 NBT staining and microscopic observation of hyphal morphology. A** NBT staining of *A. flavus* GE1 and *A. niger* GE2 with or without SynCom1 stream coinoculation. **B** Morphology of hyphae proximal and distal to the SynCom1 stream. Red arrows indicate the twisted and swelled hyphae. NBT, nitroblue tetrazolium, Bars, 10 µm.

**
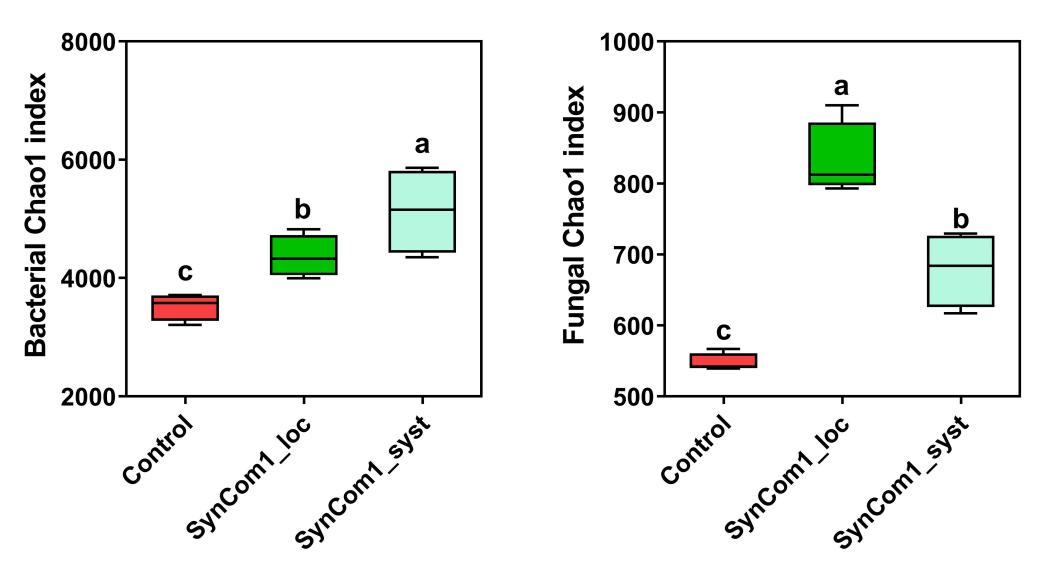
**

**Supplementary Figure 17 The bacterial and fungal Chao1 index analysis of samples from pot experiment 2.** Bacterial and fungal Chao1 index analysis of control, SynCom1_loc, and SynCom1_syst samples. Boxplots indicate median (middle line), 25th, 75th percentiles (box), and maximum and minimum values (whiskers) (n = 4 individual replicates). Different letters indicate significant differences (**P* < 0.05, one-way analysis of variance followed by Tukey’s honest significant difference test).

**
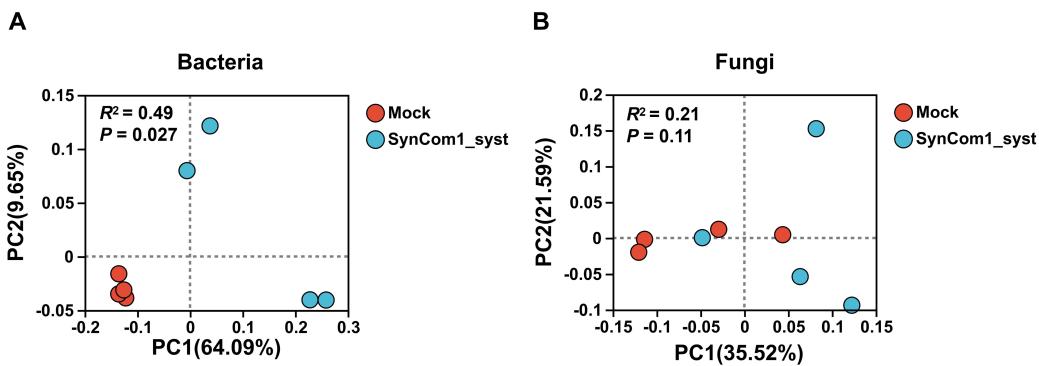
**

**Supplementary Figure 18 PCoA (based on the relative abundance of OTUs) analysis of Bray-Curtis distances of control and SynCom1_syst samples. A** PCoA (based on the relative abundance of bacterial OTUs) of Bray-Curtis distances of control and SynCom1_syst samples. **B** PCoA (based on the relative abundance of fungal OTUs) of Bray-Curtis distances of control and SynCom1_syst samples. PERMANOVA was performed using the adonis function from the R package.

**
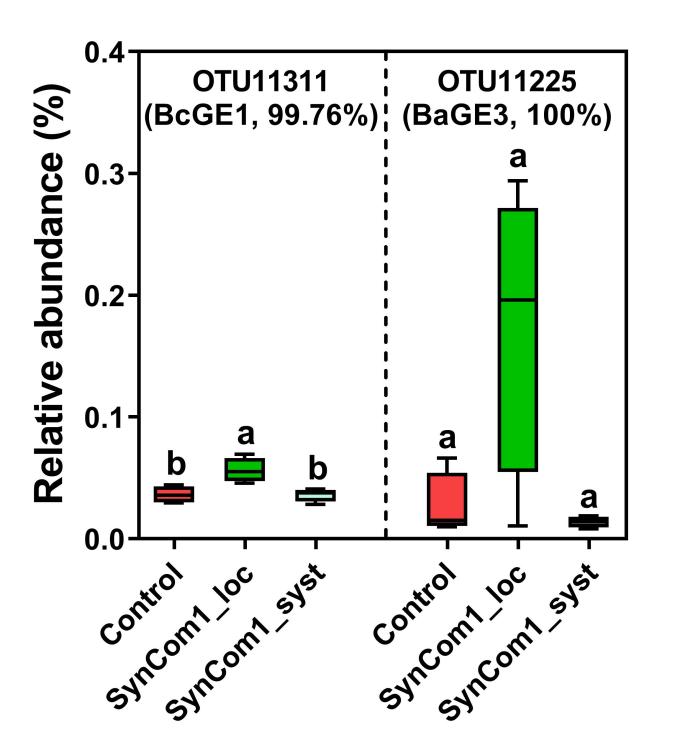
Supplementary Figure 19 The relative abundance of OTU11311 and OTU11225.** The OTU11311 and OTU11225 showed 99.77% and 100% homology to *B. cereus* GE1 and *B. altitudinis* GE3, respectively. Boxplots indicate median (middle line), 25th, 75th percentiles (box), and maximum and minimum values (whiskers) (n = 4 individual replicates). Different letters indicate significant differences (*P* < 0.05, one-way analysis of variance followed by Tukey’s honest significant difference test).

**
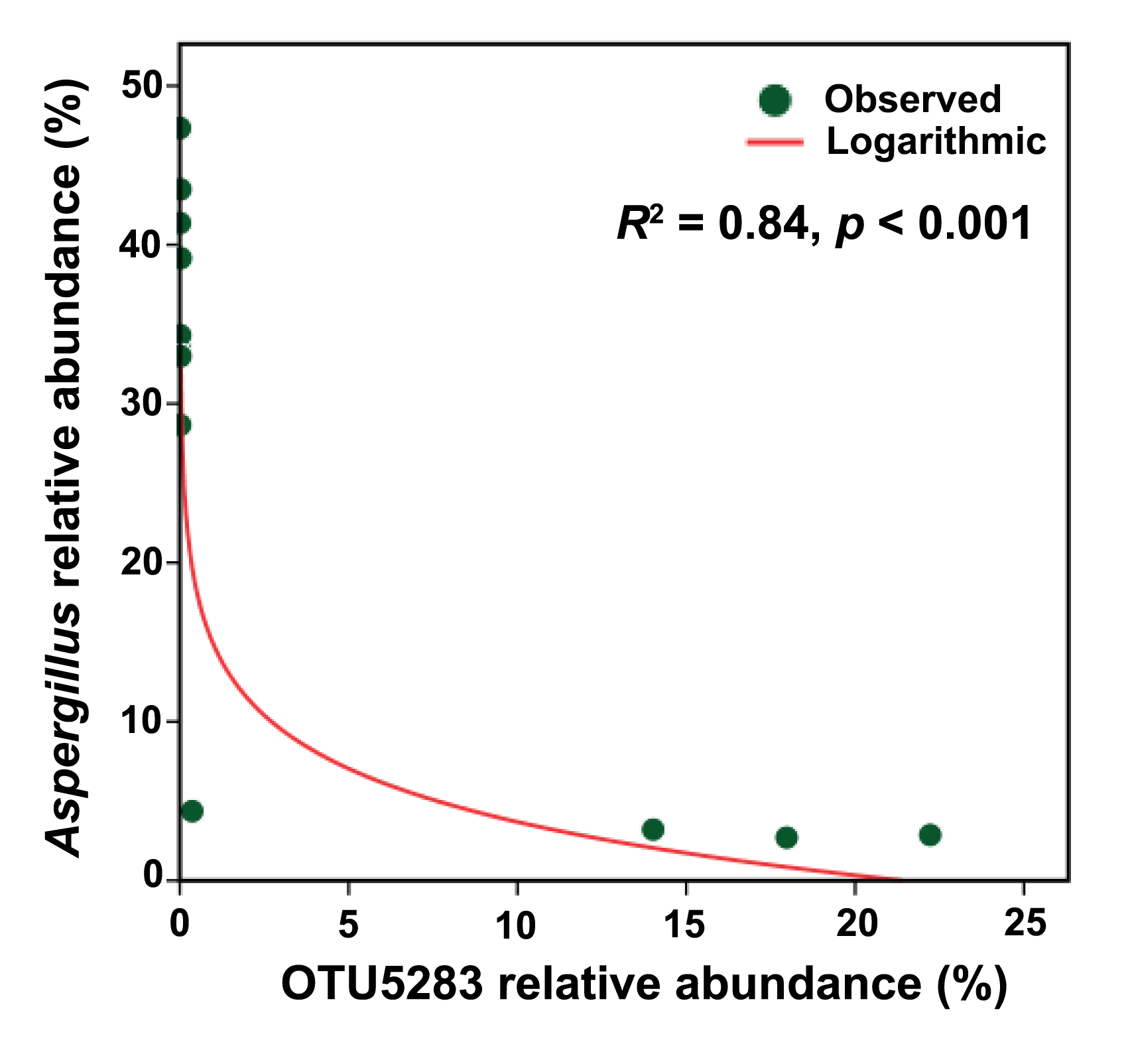
Supplementary Figure 20 Correlation relationship between relative abundance of OTU5283 and relative abundance of *Aspergillus*.**
